# Ultrasensitive chiral detection by nonlinear chiroptics in spiral plasmonic metastructures surpasses linear limits

**DOI:** 10.1126/sciadv.aed9129

**Published:** 2026-09-23

**Authors:** Yong Tan, Yufeng Cheng, Xiaolin Lu, Huatian Hu, Xiaoze Liu, Alexander Govorov, Tao Ding

**Affiliations:** ^1^ Key Laboratory of Artificial Micro/Nano Structure of Ministry of Education, School of Physics and Technology, Wuhan University, Wuhan 430072, China.; ^2^ Center for Biomolecular Nanotechnologies, Istituto Italiano di Tecnologia, Arnesano 73010, Italy.; ^3^ Department of Physics and Astronomy, Ohio University, Athens, OH 45701, USA.

## Abstract

Chiral sensing is vital in pharmaceuticals and biomedicine but is hindered by the inherently weak chiroptic signals of molecules. Conventional methods rely on tedious chromatographic techniques with expensive chiral columns. This work introduces a breakthrough in ultrasensitive chiral detection by combining nonlinear optics and tailored chiral plasmonics. We fabricate spiral gold metastructures that provide strong enhancements of both electric and superchiral near fields, which simultaneously optimize both the nonlinear conversion efficiency and dissymmetry factor, thereby generating a prominent nonlinear chiroptic response to chiral molecules. The resulting change of circularly polarized second-harmonic generation (ΔCP_SHG_) demonstrates remarkable sensitivity to chiral adsorbates, achieving a record-low detection limit of ∼11 picomolars and an outstanding figure of merit of 3260 per micromolar. A key advantage is the ability to selectively detect specific enantiomers directly within racemic mixtures, thereby dispensing with the need for preliminary chromatographic separation. This platform opens promising avenues for chiral analysis, pushing the boundaries of sensitivity, selectivity, and miniaturization for next-generation biochemical assays.

## INTRODUCTION

Chirality, a fundamental asymmetry in molecular structures, governs essential biochemical processes (*1*), drug efficacy (*2*), and material properties (*3*). However, distinguishing enantiomers remains analytically challenging because of weak chiroptic responses in conventional techniques (*4*), which suffer from low sensitivity (approximately millimolar detection limits) and lengthy measurements (*5*). Although nanophotonic approaches have substantially improved the sensitivity of chiroptic detection to extremely low concentrations (femtomolar to zeptomolar) (*6*–*10*) or even single-molecule level (*11*, *12*), the broad linewidth and large noise level of the circular dichroism (CD) have led to poor reproducibility with low figure of merit (FoM), rendering them far from practical applications (*13*–*16*). Here, the FoM can be defined as the following two equations, depending on the measurement

$$\text{FoM}=\frac{∆∆∆\lambda }{∆c\cdot \text{FWHM}}$$ (1)

$$\text{FoM}=\frac{∆\text{CD}}{∆c\cdot \sigma_{n}}$$ (2)

where Eqs. 1 and 2 are applicable for sensing based on spectral shift (*17*–*25*) and intensity change (*9*, *26*–*28*), respectively. Δ∆∆λ/∆*c* and ∆CD/∆*c* is the change rate of spectral shift dissymmetry (ΔΔλ) and CD intensity with analytes’ concentration, respectively. FWHM (full width at half maximum) is the CD spectra linewidth and σ_n_ is the noise level of CD spectra.

Although surface-enhanced Raman scattering offers exceptionally high sensitivity for detecting chiral molecules (*29*, *30*), its reproducibility is often compromised because of its strong dependence on the precise positioning of molecules within plasmonic hotspots. In contrast, nonlinear chiroptic sensing based on surface symmetry breaking is an inherently coherent process, which provides a more quantifiable and stable signal directly correlated with the analyte’s adsorption. This technique also suppresses fluorescence background and can probe molecular conformations inaccessible to linear chiroptic spectroscopy (*31*, *32*). However, nonlinear optical effects are inherently weak and become even fainter when combined with small chiral asymmetries (*33*).

Nonlinear plasmonics (*34*), due to the enhanced electric (*E*) near-field (enhancement factor up to ∼1000 times) in the nanocavity, can substantially enhance the nonlinear processes (*35*, *36*), including the second-harmonic generation [SHG; $\propto E{\left(\omega \right)}^{4}$] and third-harmonic generation [THG, $\propto E{\left(\omega \right)}^{6}$] at the nanoscale (*37*–*43*). With this quadratic and cubic enhancement, nonlinear plasmonics has demonstrated substantial potential for advanced optical sensing and bioimaging (*44*–*47*). The FoM of the SHG plasmonic sensors can substantially exceed that of its linear counterpart due to their different scaling relationships as the former follows ∼*Q*(ω)^2^*Q*(2ω)/*V*_m_ (*48*), while the latter follows ∼*Q*/*V*_m_ (*49*), where *Q* represents the quality factor of the plasmonic nanocavity and *V*_m_ denotes its mode volume. The potential of nonlinear chiral nanophotonic structures have been demonstrated through various proposed (*50*, *51*) and developed (*52*–*57*) designs in the past decade, but their application in chiral molecular sensing via SHG remains challenging. Although SHG-based chiral plasmonic sensing has been established on the basis of the change of both reflectivity and chiroptic properties (*58*), the detection method is not straightforward for quantitative analysis. This is primarily because the nonlinear chiroptic sensing lacks mature theoretical frameworks (*59*–*61*) and suitable material systems that exhibit both strong nonlinearity and notable chiral response (*62*–*67*). Moreover, the chiral background signals of the chiral plasmonic structures often overwhelm the chiral response of the molecules (*27*, *28*), making the extraction of meaningful information and quantitative analysis difficult.

**Fig. 1.**
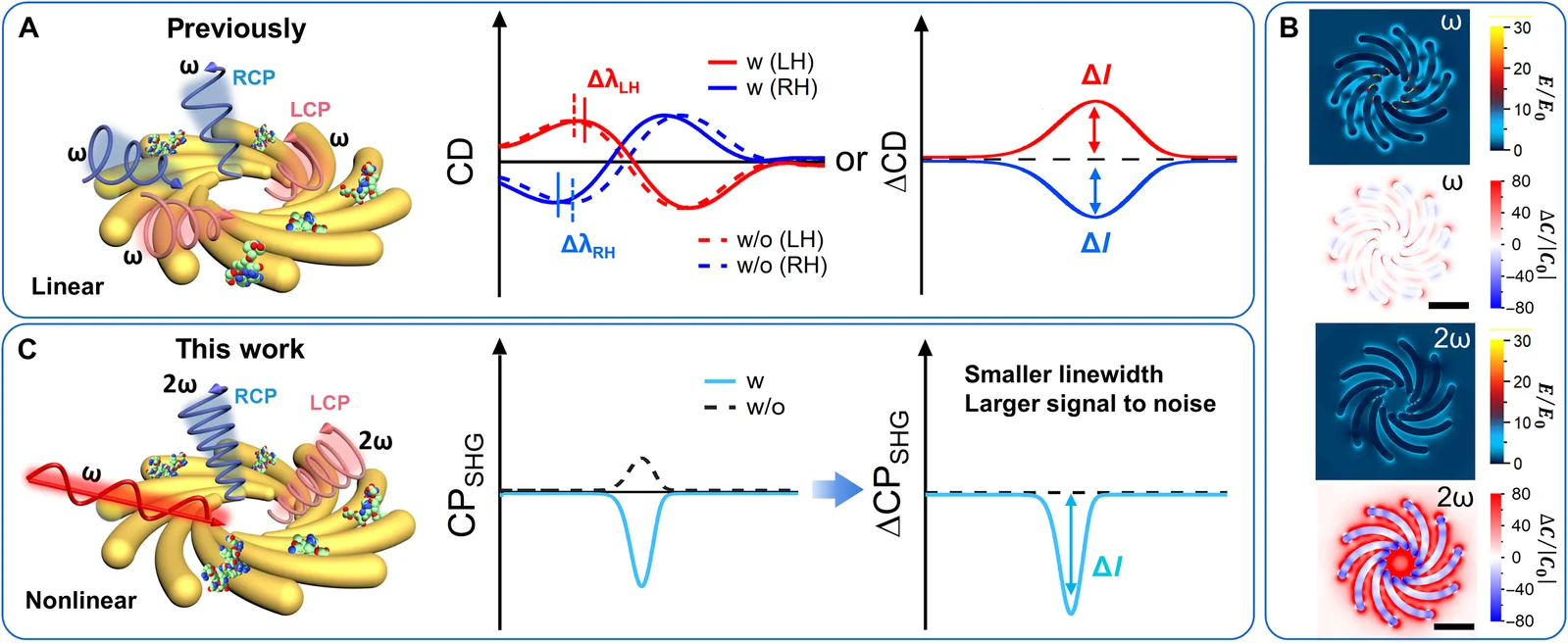
Chiral plasmonic sensing based on LCD and NLCD. (**A**) Previously demonstrated concept of chiral plasmonic sensing based on LCD. (**B**) *E*-field (*E*/*E*_0_) and optical chirality difference (∆*C*/|*C*_0_|) enhancement of spiral Au metastructure at ω and 2ω frequency. *E*_0_ and *C*_0_ represent the optical near-field without any structures. Scale bars, 500 nm. (**C**) Chiral plasmonic sensing based on CP_SHG_ demonstrated in this work.

Excellent nonlinear chiroptic sensing relies on simultaneously enhancing two inherently weak effects of nonlinearity and chirality. The former typically demands ultrasmall optical cavities, while the latter requires larger-scale chiral superstructures. Here, we fabricate spiral plasmonic metastructures with desired handedness via vector beam writing to achieve enhanced chiral sensing based on their amplified nonlinear chiroptic response. This metastructure resolves the paradox by incorporating both small gaps and large chiral domains, enabling a balanced and effective enhancement for chiral sensing. Rather different from conventional linear CD (LCD), which is mostly based on the shift of a broad CD peak of plasmons (*20*) or the change in its intensity (Fig. 1A) (*27*), we focus on its nonlinear chiroptic response based on the circularly polarized SHG (CP_SHG_ = *I*_*2*ω,LCP_ − *I*_*2*ω,RCP_). Because of the extremely large *E*-field enhancement (∼85) at fundamental frequency (ω) between the spiral arms and the optical chirality difference (∆*C* = *C*_LCP_ − *C*_RCP_) enhancement (∼400) at the second-harmonic frequency (2ω) around the spiral arms (Fig. 1B), it presents balanced nonlinear chiroptic performance with both high conversion efficiency (η = *I*_*2*ω_/*I*_ω_) and large dissymmetry [*g*-factor = 2(*I*_LCP_ − *I*_RCP_)/(*I*_LCP_ + *I*_RCP_)]. Hence, we can use the change of circularly polarized harmonic emission (ΔCP_SHG_) as a primary sensing readout to discriminate and quantify the chiral substances around the surfaces (Fig. 1C), enabling quantitative assays and enantiopurity readout in racemates. The limit of this nonlinear detection (LoD_NL_) can reach as low as ∼11 pM with FoM up to 3260 μM^−1^, which outperforms the state-of-the-art linear plasmonic sensing system (*19*, *23*, *26*), shifting the paradigm for chiral sensing.

## RESULTS

**Fig. 2.**
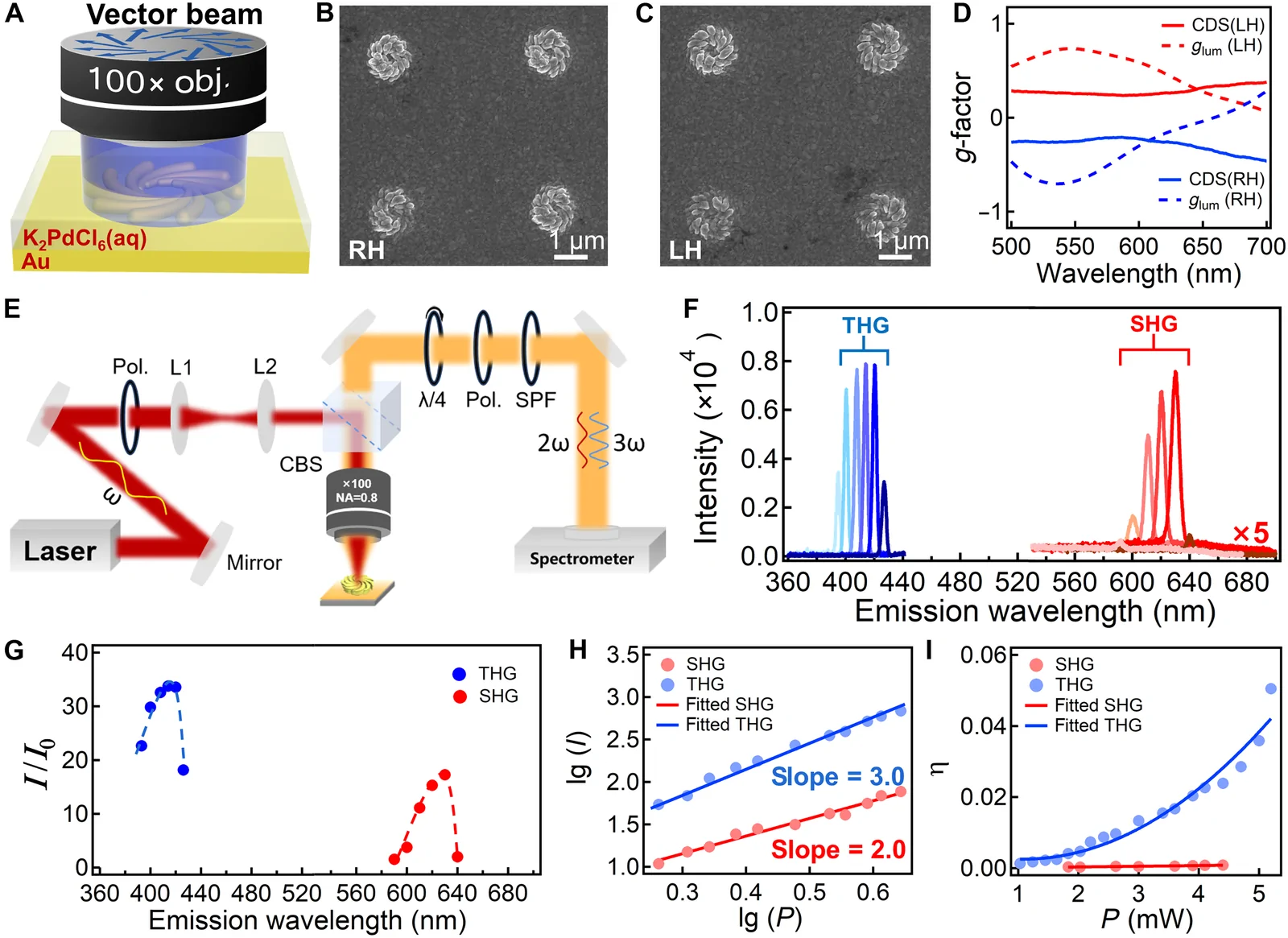
Fabrication of spiral Au metastructures and their linear and nonlinear optic responses. (**A**) Schematic of vector beam induced growth. (**B** and **C**) Scanning electron microscopy (SEM) images of (B) RH and (C) LH spiral metastructure arrays. (**D**) *g*-factor spectra of chiral scattering and CPL of LH and RH spiral Au metastructures. (**E**) Schematic of SHG and THG measurement setup. Pol., linear polarizer; L1/L2, beam expander; CBS, 50/50 cube beam splitter; λ/4, quarter-wave plate; SPF, 700-nm short-pass filter. (**F**) SHG and THG spectra of spiral Au metastructures excited with varied FW from 1180 to 1280 nm. (**G**) Enhancement factor of the SHG and THG intensities of the spiral Au metastructures. *I* and *I*_0_ refer to the SHG/THG intensities of spiral Au metastructures and Au films, respectively. (**H**) Logarithmic plots of SHG and THG intensity versus incident power. The red and blue solid lines represent the linear fitting of SHG and THG intensities with slopes of 2.0 and 3.0, respectively. (**I**) Correlation between the SHG/THG conversion efficiency and the excitation power at FW. The red and blue solid lines represent the linear and quadratic power law fitting of SHG and THG intensities, respectively.

The spiral plasmonic metastructures were fabricated via direct laser writing with spiral vector beams (Fig. 2A) (*20*, *68*), which can controllably generate arrays of left-handed (LH) and right-handed (RH) enantiomers on Au substrate (Fig. 2, B and C). They show distinct chiral scattering of left-/right-circularly polarized (LCP/RCP) light (fig. S1, A and B), which generates a scattering *g*-factor of 0.45 ± 0.05 (solid lines in Fig. 2D). Excitation with a continuous-wave (CW) laser (447 nm) can also generate plasmon photoluminescence (PL), which presents different emission intensities of LCP and RCP light (fig. S1, C and D). The *g*-factor (*g*_lum_) of circularly polarized luminescence (CPL) can reach up to ±0.73 at ∼550 nm but decrease below ±0.45 over 600 nm (dashed lines in Fig. 2D). These linear chiroptic responses all show broad emission peaks with very low quantum efficiency (∼0.2%) due to the damping of plasmons.

We further performed nonlinear optical measurements of these spiral metastructures with the excitation of a femtosecond-pulsed laser (Fig. 2E). We varied the fundamental wavelength (FW) from 1180 to 1280 nm and found that both the SHG and THG signals can be detected at the wavelengths of 620 ± 20 and 410 ± 10 nm, respectively (Fig. 2F). The strongest SHG and THG peaks are at 632 and 421 nm, which exhibit 17- and 33-fold enhancements, respectively, as compared to the evaporated gold films (thickness, 100 nm) (Fig. 2G). This is due to the plasmon resonances at the FW of 1260 nm (fig. S2), which yields the largest enhancement of SHG and THG emission. Thus, we adopt 1260 nm as the FW excitation in the following experiments unless stated otherwise.

The power dependence of SHG and THG signals shows superlinear correlation with slopes of 2.0 and 3.0, respectively (Fig. 2H), which further confirms that these peaks are from SHG and THG signals. The nonlinear conversion efficiency shows linear dependence for SHG and nonlinear dependence for THG, which again verifies their nonlinear optical processes (Fig. 2I and Materials and Methods for the calculation details). The THG efficiency typically surpasses that of SHG since the latter is constrained by surface symmetry breaking, whereas the former is a volumetric process. Although the surface exhibits the strongest field, its extremely thin region contributes less than the largely enhanced near-field volume, where substantial field strength and favorable phase matching dominate the total THG emission. Moreover, the different nonlinear scaling with plasmonic enhancement for SHG and THG at FW also renders much higher conversion efficiency for THG in the plasmonic nanostructures (*42*).

**Fig. 3.**
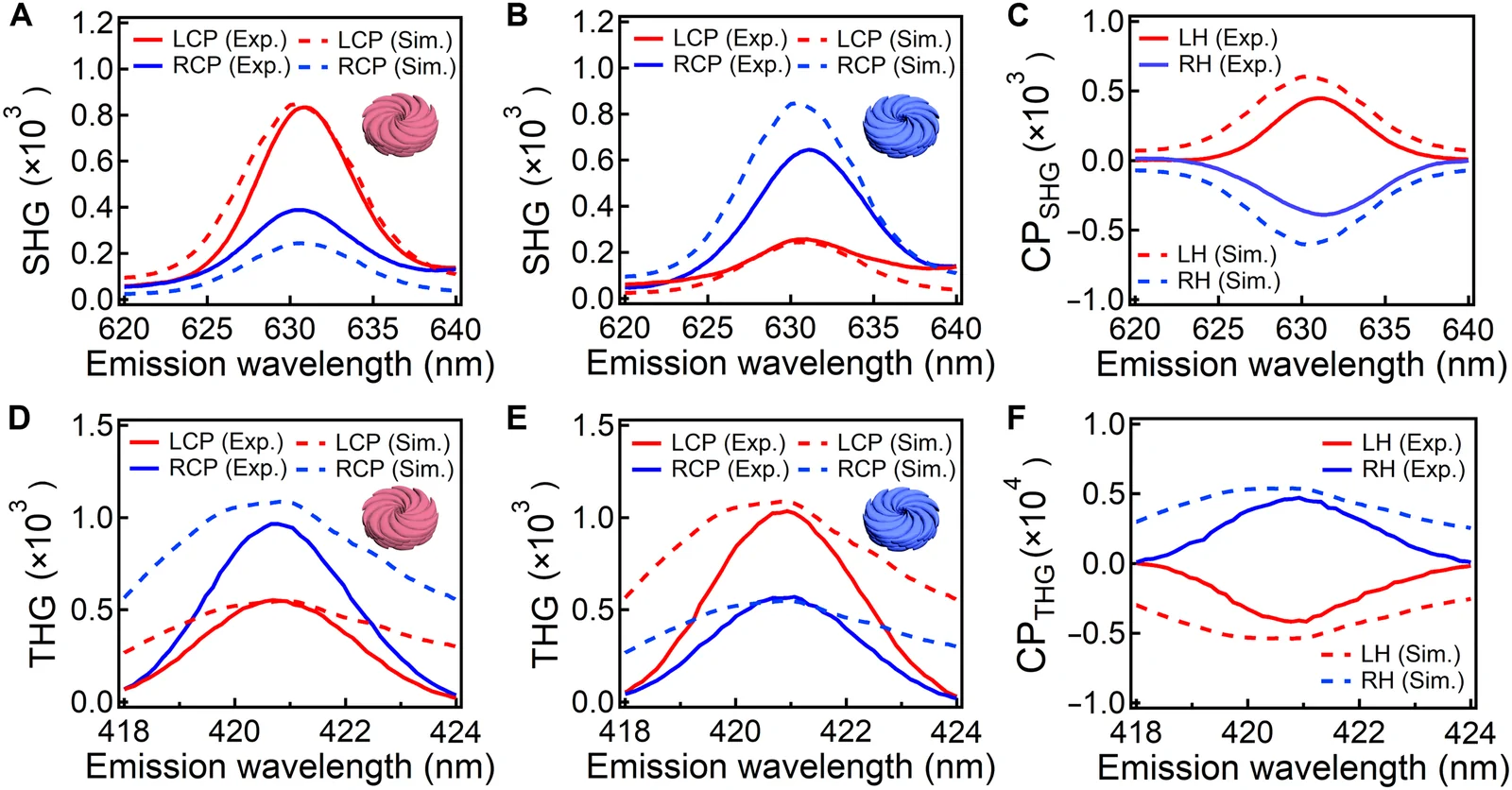
Nonlinear chiroptic properties of spiral Au metastructures. (**A** to **C**) Chiral SHG spectra of (A) LH and (B) RH spiral Au metastructures and their (C) CP_SHG_ spectra. (**D** to **F**) Chiral THG spectra of (D) LH and (E) RH spiral Au metastructures and their (F) CP_THG_ spectra. Insets of (A), (B), (D), and (E) are the schematics of LH and RH spiral Au metastructures. Solid lines are experimental results, and dashed lines are simulated spectra with arbitrary units.

The nonlinear chiroptic measurement is performed with the same FW excitation but with a properly aligned λ/4 wave plate and polarizer (±45°) placed before the spectrometer. We can see a distinct difference for the LCP and RCP emission of SHG (Fig. 3, A and B) and THG (Fig. 3, D and E) signals from LH and RH spiral Au metastructures, which generates CP_SHG_ of ±450 and CP_THG_ of ±4600 (Fig. 3, C and F), while for achiral plasmonic structures, negligible difference can be found (fig. S3). Simulations of the chiral SHG and THG spectra generated by these spiral Au metastructures (dashed lines in Fig. 3) show excellent agreement with the experimental results, further validating the nonlinear chiroptic measurements.

The *g*-factors and conversion efficiencies of CPL, SHG, and THG are summarized in Table 1. Note that the *g*_SHG_ is slightly larger than both *g*_THG_ and *g*_lum_ mainly because it is highly sensitive to the surface symmetry where the chiral feature is prominent. While for achiral structures, the *g*-factors appear almost the same as evaporated Au films. For the conversion efficiency of spiral Au, the SHG efficiency is almost the same as CPL, but the THG is an order of magnitude higher. Nevertheless, all of them are much larger than those of the achiral Au and Au films due to local enhancement of *E*-field at the nanogaps between the spiral arms.

**Table 1.** Summary of g-factors and conversion efficiencies of CPL, SHG, and THG of different Au samples. The data shown in this table are extracted from Figs. 2 (D and I) and 3 (C and F) and fig. S3.

Samples	*g*_CPL_	*g*_SHG_	*g*_THG_	η_CPL_	η_SHG_	η_THG_
Spiral Au	0.7	0.75	0.6	2.3 × 10^−3^	8.0 × 10^−4^	5.0 × 10^−2^
Achiral Au	0.021	0.037	0.028	1.4 × 10^−3^	2.2 × 10^−4^	1.2 × 10^−2^
Au films	0.007	0.015	0.057	1.1 × 10^−3^	4.6 × 10^−5^	1.2 × 10^−3^

Another noteworthy difference between CP_SHG_ and CP_THG_ is that their enantiomers show opposite signs of CP spectra (Fig. 3, C and F). In the CP_SHG_ spectra, the LH structures show positive CP_SHG_, and RH structures show negative CP_SHG_ (Fig. 3C), which is consistent with the trend of CPL spectra (Fig. 2D). While for the CP_THG_ spectra, the chiroptic response of CP_THG_ is reversed (Fig. 3F). Such a chiral reversion is largely related to the optical chirality (*C*) at the emission frequency, which determines the asymmetric emission rate of SHG and THG. As the circular differential scattering (CDS), CPL, and CP_SHG_ reflect the intrinsic electronic/optical chirality linked to electric and magnetic dipole responses at 2ω, they share the same sign of chiroptic response, while for CP_THG_, the optical chirality at 3ω appears as a complementary pattern (fig. S4), thereby inverting the sign of CP_THG_.

The nonlinear chiroptic response of these spiral Au metastructures can be adjusted by controlling the growth time (text S3). With increasing irradiation time, the height of the spiral Au increases, and the spiral arms gradually merge together, leading to less pronounced chiral features. As a result, both the CP_SHG_ and CP_THG_ first increase with irradiation time and then decrease (figs. S5 and S6). The *g*_SHG_ reaches the maximum of 0.75 at a height of 500 nm, while the *g*_THG_ reaches the minimum of −0.6 (fig. S6). This is mainly because prolonged growth can merge the spiral arms, and this merging leads to the closure of the nanogaps within the metastructures, which reduces both the local *E*-field and optical chirality enhancement.

**Fig. 4.**
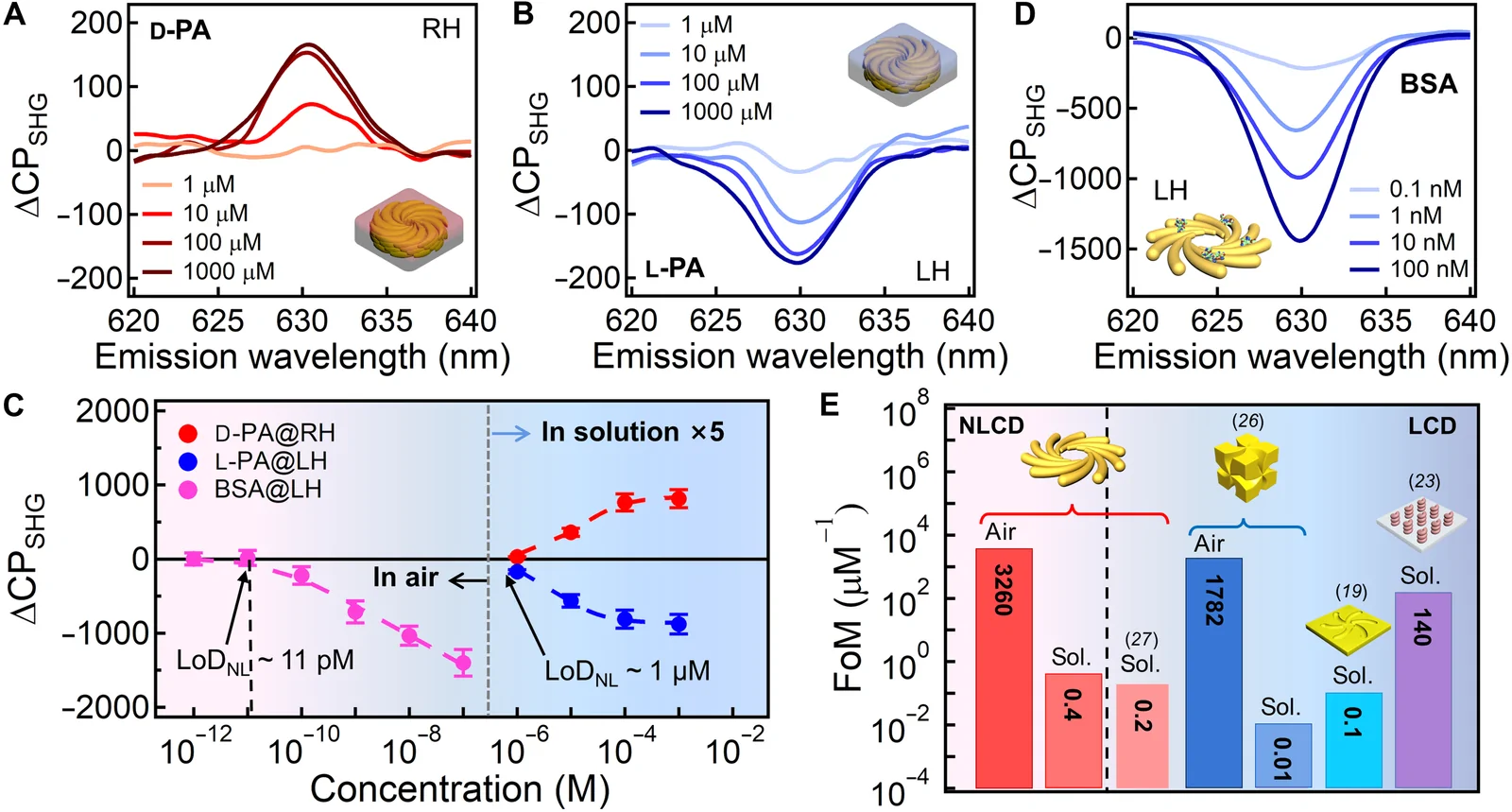
Nonlinear chiroptic sensing based on spiral Au metastructures. (**A** and **B**) ΔCP_SHG_ spectra of (A) RH and (B) LH spiral Au metastructure immersed in d-PA and l-PA solutions of different concentration. (**C**) Change of ΔCP_SHG_ maximum value with concentration of d-PA, l-PA, and l-BSA detected with RH/LH spiral Au metastructures. Error bars are standard deviation of three repeated measurements (fig. S17). (**D**) ΔCP_SHG_ spectra of l-BSA adsorbed on LH spiral Au metastructures with different concentrations. All the CP_SHG_ spectra were taken in air at ambient condition. (**E**) FoM of different chiral plasmonic sensors in the linear and nonlinear optical regimes (*19*, *23*, *26*, *27*).

We use the spiral Au metastructures with optimized nonlinear chiroptic performance for chiral sensing applications. Specifically, we first measured the CP_SHG_hybrid_ from the spiral Au enantiomers in the presence of amino acid [phenylalanine (PA)] solutions of different concentrations and then subtracted the chiral SHG signals without the molecules (CP_SHG_bkg_) to get the net CP_SHG_ (ΔCP_SHG_) induced by l-/d-PA only. We found that d-PA induced a marked change in the CP_SHG_ of RH spiral Au and l-PA induced a marked change in the CP_SHG_ of LH spiral Au. In contrast, their respective enantiomers had a much weaker effect on the CP_SHG_ of RH and LH spiral Au (figs. S7 and S8), suggesting enantioselective sensing via the CP_SHG_ channel. Note that at a concentration of 10 μM, they can even reverse the sign of CP_SHG_, suggesting strong modification of $\chi_{\text{surf}}^{\prime \prime }$. As a result, their ΔCP_SHG_ spectral intensity shows a monotonic increase/decrease with increasing concentration of enantiomers for RH/LH spiral Au, respectively (Fig. 4, A and B). As a control, we measured the ΔCP_SHG_ response of RH and LH spiral Au in the presence of achiral molecules (cysteamine, ∼1 mM) and found that they show nearly zero ΔCP_SHG_ intensities (fig. S9), suggesting validity of the nonlinear chiroptic measurements. We also performed the chiral sensing from the CP_THG_ channel with the same measurement procedure. Although ΔCP_THG_ shows linear correlation with concentration, it cannot distinguish the enantiomers (fig. S10). This is mainly because the THG signal scales with the cube of the fundamental electric field and is sensitive to phase matching/coherence over the entire volume. Thus, the small perturbation of enantiomers adsorbed on the surface is not sufficient to alter the sign of CP_THG_, as it does in the SHG process. Further, we used achiral Au as a contrast for the chiral sensing, and no obvious ΔCP_SHG_ signals could be detected, resulting in poor sensing capability for the chiral molecules (fig. S11).

We perform theoretical analysis to rationalize these observations. Initially, we have the intrinsic CP_SHG_ of the spiral Au metastructures as the background, which is expressed as

$$\begin{array}{c} {\text{CP}}_{\text{SHG}\_\text{bkg}}=I_{\text{LCP}}^{\text{SHG}}-I_{\text{RCP}}^{\text{SHG}}\propto \int dS\cdot \\ \left({|\overset{=}{G_{\text{LCP}}}\left(r,r^{\prime }\right)\cdot p^{\text{SHG}}\left(r^{\prime }\right)|}^{2}-{|\overset{=}{G_{\text{RCP}}}\left(r,r^{\prime }\right)\cdot p^{\text{SHG}}\left(r^{\prime }\right)|}^{2}\right) \end{array}$$ (3)

Here, dS is differential detection cross section at r. $p^{\text{SHG}}\left(r^{\prime }\right)$ is the second-harmonic polarization generated by the interplay of local electric field with the second-order surface susceptibility tensor of Au, which can be expressed as $p^{\text{SHG}}\left(r^{\prime }\right)\propto \chi_{\text{surf}}^{\prime \prime }\left(r^{\prime }\right)E\cdot E$ in general. Its detailed components can be divided into the surface and bulk contributions as shown in Eqs. 15 to 17. The local *E*-field in Eqs. 15 to 17 was calculated by a linearly polarized light excitation. The second-harmonic polarizations (regarded as microscopic emitters) then emitted out through the spiral Au metastructures, which serve as a nanoantenna. For a second-harmonic point dipole in a chiral nanostructure, the intrinsic and extrinsic chirality will dress the emitted photons with certain circular polarization through a circular polarization–encoded Green’s function ($G_{\text{LCP}}$ and $G_{\text{RCP}}$) (*69*). Here the $G_{\text{LCP},\text{RCP}}$ elements from complex geometries can be calculated by decomposing the Poynting vector into LCP and RCP components in the far field (see details in Materials and Methods). The integral is performed to consider the power in the collection cone.

When chiral molecules are introduced to the system, they can modify this CP_SHG_ response through surface adsorption, which strongly alters $\chi_{\text{surf}}^{\prime \prime }$. Thus, the CP_SHG_ becomes

$$\begin{array}{c} {\text{CP}}_{\text{SHG}\_\text{hybrid}}\propto \int dS\cdot \\ \left({|\overset{=}{G_{\text{LCP}}}\left(r,r^{\prime }\right)\cdot p_{\text{eff}}^{\text{SHG}}\left(r^{\prime }\right)|}^{2}-{|\overset{=}{G_{\text{RCP}}}\left(r,r^{\prime }\right)\cdot p_{\text{eff}}^{\text{SHG}}\left(r^{\prime }\right)|}^{2}\right) \end{array}$$ (4)

where $p_{\text{eff}}^{\text{SHG}}\left(r^{\prime }\right)\propto \chi_{\text{eff}}^{\prime \prime }\left(r^{\prime }\right)E\cdot E$. Here, $\chi_{\text{eff}}^{\prime \prime }=\chi_{\text{surf}}^{\prime \prime }+\theta \cdot \chi_{\text{mol}}^{\prime \prime }$. Here, $\chi_{\text{mol}}^{\prime \prime }$ is the tensor component of intrinsic chiral response from the second-order nonlinear susceptibility of chiral molecules, and θ is the surface coverage (density) of chiral molecules. Since the $\chi_{\text{mol}}^{\prime \prime }$ is very small for molecules, the direct molecular contribution to SHG can be ignored. Thus, we can further derive ΔCP_SHG_ as

$$\begin{array}{c} ∆{\text{CP}}_{\text{SHG}}={\text{CP}}_{\text{SHG}\_\text{hybrid}}-{\text{CP}}_{{\text{SHG}}_{\text{bkg}}}\propto \int dS\cdot \\ 2\mathrm{\theta Re}\left[\chi_{\text{surf}}^{\prime \prime }\cdot \chi_{\text{mol}}^{\prime \prime }\right]\left({|\overset{=}{G_{\text{LCP}}}\left(r,r^{\prime }\right)|}^{2}-{|\overset{=}{G_{\text{RCP}}}\left(r,r^{\prime }\right)|}^{2}\right){|E|}^{4} \end{array}$$ (5)

This Eq. 5 is the master equation, indicating that the SHG chiral sensing signal (ΔCP_SHG_) is linearly correlated to the chiral response of the second-order nonlinear susceptibility of the plasmonic structures ($\chi_{\text{surf}}^{\prime \prime }$) and the molecules ($\chi_{\text{mol}}^{\prime \prime }$) as well as their surface coverage density (θ), which is amplified in the fourth power of electric field intensity at the FW. The difference between $G_{\text{LCP}}$ and $G_{\text{RCP}}$, which correlates to the optical chirality difference (∆*C*) at 2ω, also contributes to the enhancement of ΔCP_SHG_. Thus, either zero $\chi_{\text{surf}}^{\prime \prime }$ (achiral Au) or zero $\chi_{\text{mol}}^{\prime \prime }$ (achiral molecule) will lead to zero ΔCP_SHG_, which agrees with the experimental observation (figs. S9 and S11). It has to be noted that Eq. 5 is for a phenomenological analysis and the rigorous calculation should be done with careful treatment of the second-harmonic polarizations following Eqs. 15 to 17.

Because of the complex and heterogeneous nature of the spiral Au metasurfaces, a Temkin-II isothermal adsorption model is applied for protein adsorption (*70*), where $\theta \propto \text{ln}\left(1+K_{0}c\right)$ (see text S5 for detailed clarification). Combined with Eq. 5, we find a linear correlation of $∆{\text{CP}}_{\text{SHG}}\propto \theta \propto \text{ln}\left(c\right)$, which is justified by the experimental calibration curves shown in Fig. 4C. Fitting with the Temkin model suggests a detection limit of 1.0 μM (blue and red dashed lines in Fig. 4C), which outperforms conventional CD (∼100 μM) by two orders of magnitude and an order of magnitude higher than that (∼10 μM) detected by the same spiral Au metastructures but in the linear optical regime (*20*, *27*).

For the abovementioned chiral sensing, the measurements were taken immediately after the spiral Au metastructures were immersed in the aqueous solution of PA. However, when the spiral Au metastructures were incubated with the PA solution for a prolonged period time (40 min) when sufficient adsorption of PA happens, we found an inversion of the sign and a marked increase in the CP_SHG_ signals even after removing the solution (fig. S12). This observation provides crucial evidence that it is the change of the chiral surface susceptibility of Au ($\chi_{\text{surf}}^{\prime \prime }$) via the adsorption of chiral molecules ($\chi_{\text{mol}}^{\prime \prime }$) that induces this strong modification of ΔCP_SHG_ with the amplification of asymmetric *E*-field (Eq. 5).

Since the chiral SHG signals are highly sensitive to surface bound molecules, the actual number of molecules that contribute to the SHG could be even smaller. To evaluate this, we measured the ΔCP_SHG_ signals with thiolated protein molecules [bovine serum albumin (BSA)], which can be stably immobilized on the spiral Au metastructures in air without the presence of liquid background. Here, as BSA is LH and LH spiral Au is optimized for the sensing of L-type enantiomers (Fig. 4B), we choose LH spiral Au metastructures for the sensing of BSA. As the dried BSA shows strong chemisorption via Au─thiol bonding and large chiral domains of intensive secondary structures such as α helices and β sheets, they can effectively couple to the plasmonic superchiral fields, which induces a much larger change in the surface susceptibility of Au ($\chi_{\text{surf}}^{\prime \prime }$). The orientation of large secondary structures currently cannot be easily controlled experimentally; however, they do play an important role in the chiral SHG response (fig. S13). Nevertheless, even at a concentration as low as 0.1 nM, the presence of adsorbed BSA can still be detected with ΔCP_SHG_ of ∼200 (Fig. 4D and figs. S14 to S17). The number of BSA molecules that contributed to such a large ΔCP_SHG_ is ∼1.5 × 10^6^ as determined by quartz microbalance (fig. S18), but the actual number is hard to quantify via simple Temkin model due to the presence of intricate nanogaps within the spiral Au metastructures, which may lead to different packing densities as compared to a flat Au surface. Nevertheless, the dried BSA protein shows ∼100 times larger CP_SHG_ enhancement than PA solution, suggesting that surface binding and molecular size/organization are crucial for maximizing the chirality transfer effect especially in the nonlinear regime. In this particular case, the superchiral fields from spiral metastructure couple to BSA’s extended chiral architecture, which creates a large and stable surface dipole asymmetry, thereby modifying the effective surface $\chi_{\text{surf}}^{\prime \prime }$ for enhanced ΔCP_SHG_. The nonlinear sensing LoD_NL_ for BSA can reach ∼11 pM (pink dashed line Fig. 4C), while its linear sensing LoD_L_ is only ∼1 nM (fig. S19), suggesting that the nonlinear CD (NLCD) is superior to the LCD. As compared to other advanced chiroptic sensing systems such as helicoid nanoarrays (LoD_L_ ∼ 114 pM) (*26*), this NLCD is an order of magnitude lower.

We evaluate FoM of various chiral plasmonic sensors based on Eq. 2 (Fig. 4E and fig. S20) and find that this spiral plasmonic sensor shows nonlinear FoM up to ∼3260 μM^−1^, while its linear FoM is only 0.02 μM^−1^ (fig. S19). Other superior linear plasmonic sensors such as helicoid arrays (*26*) and Ag nanohelicoids (*23*) only present FoM of 1780 and 140 μM^−1^, respectively. Other structures such as shuriken (*19*) show even smaller FoM with values less than 1. It is clear from this comparison that this type of nonlinear plasmonic sensor is on the top of superior chiral sensing systems.

**Fig. 5.**
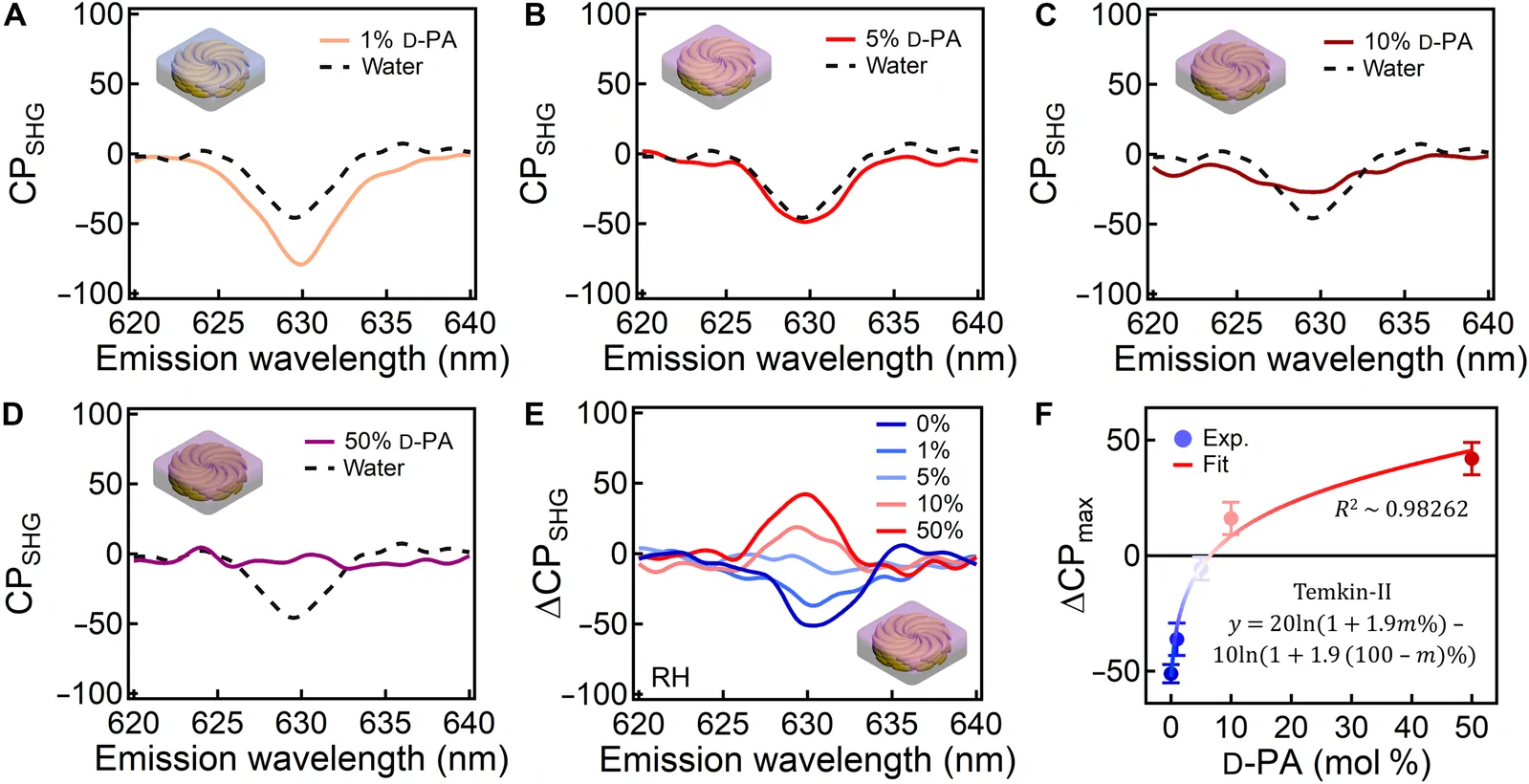
Sensing performance of enantiomer mixture based on RH spiral Au metastructures. (**A** to **D**) CP_SHG_ spectra of RH spiral plasmonic metastructures immersed in the enantiomer mixture with different molar ratios of d-PA: (A) 1%, (B) 5%, (C) 10%, and (D) 50%. The total concentration of the mixture is 100 μM. The dashed lines are the CP_SHG_ spectra of RH spiral Au metastructure in DI water. (**E**) ΔCP_SHG_ spectra of RH spiral Au metastructures immersed in the enantiomer mixture with different molar ratios of d-PA. (**F**) Change of ΔCP_max_ with increasing molar ratio of d-PA. Dots are experimental data, and solid line is their bimodel Temkin fitting curve.

The perfect mirror symmetry in our observations (LH structures recognizing l-PA and RH structures recognizing d-PA) provides strong evidence for chiroptic recognition although they appear on notable different scales. The nonlinear optical process of SHG enhances these subtle chiral interactions, making them detectable through ΔCP_SHG_ measurements. With this enantioselective sensing mechanism, it can also quantify the purity of enantiomers (Fig. 5). Specifically, we measured the CP_SHG_ spectra of the RH spiral Au metastructures in the mixture of d-/l-PA solutions (solid lines in Fig. 5, A to D) and used water as a background reference (dashed lines in Fig. 5, A to D), from which we obtain ΔCP_SHG_ spectra (Fig. 5E). We found that as the molar ratio of d-PA increases in the solution of l-PA, the ΔCP_SHG_ curve starts to flip from negative to positive when the ratio reaches 5%. The correlation between the peak intensity of ΔCP_SHG_ and the molar ratio of d-PA fits nicely with bimodel Temkin adsorption (Fig. 5F)

$$∆{\text{CP}}_{\text{SHG}}=q_{1}\text{ln}\left(1+K_{0}\mathrm{cm}\%\right)-q_{2}\text{ln}\left(1+K_{0}c\left(100-m\right)\%\right)$$ (6)

where m% is the molar ratio of d-PA and (100−m)% is the molar ratio of l-PA in solution. *c* is the total concentration of the PA (100 μM). *q*_1_ and *q*_2_ are the fitting coefficients.

This bimodel Temkin adsorption can serve as a calibration curve for the quantification of enantiomer purity. When the molar ratio of d-PA reaches 50%, a racemic mixture is obtained which shows zero LCD response (fig. S21A). In the nonlinear optical regime, however, the RH spiral Au metastructures can selectively couple to the d-PA from the racemate mixture, leading to a pronounced positive ΔCP_SHG_ response and vice versa for LH spiral Au metastructures (fig. S21, B, C, and F). Their magnitude agrees with the averaged ΔCP_SHG_ of the enantiomers when they are measured separately (see fig. S21, D to F, and text S7 for detailed clarification), suggesting that no separation is needed for the accurate quantification of enantiomer purity. This is of immediate implication for pharmaceutical and food industries where the enantiomer purity is crucial for safety and quality control.

## DISCUSSION

In summary, we have demonstrated the chiral molecule detection by harnessing the untapped potential of nonlinear chiral plasmons. By designing spiral plasmonic metastructures, we realize giant nonlinear optical enhancements of SHG and THG with balanced conversion efficiency and dissymmetry factors (*g*-factors), which are related to large *E*-field enhancement in the nanogap of spiral arms at FW and superchiral field around the spiral metastructures at the second-harmonic frequency. We further realized label-free biomolecule detection including amino acids and proteins with ΔCP_SHG_ spectroscopy, which shows LoD_NL_ of ∼11 pM for adsorbed BSA proteins with FoM up to ∼3260 μM^−1^, rendering it a top record among the state-of-the-art chiral plasmonic sensors (Table 2). They can be further applied to enantiomer discrimination in racemates, where the RH and LH spiral Au metastructures can selectively enhance one enantiomer over the other without chiral separation, eliminating the need for tedious chromatography. By merging superchiral near-fields with giant nonlinear susceptibilities ($\chi^{\prime \prime }$), this work bridges fundamental nano-optics with practical analytical chemistry and transforms chiral sensing from cumbersome (high-performance liquid chromatography) to ultrafast, sensitive, and miniaturized nanodevices.

**Table 2.** Comparison of the sensing performance of different chiral plasmonic sensors.

Material/structure	Method	LoD	FoM^*^/μM^−1^	Ref.
Au shuriken metasurface	LCD	435 μM (EPSPS)†	0.1	(*19*)
Ag nanohelicoids	LCD	100 pM (l-/d-lysine)	140‡	(*23*)
Au helicoid two-dimensional arrays	LCD	114 pM (miR-21)	1782	(*26*)
Au hole-disk arrays	VCD	50 μM (thalidomide)	8 × 10^−3^	(*72*)
Spiral Au metastructure	NLCD	11 pM (BSA)	3260§	This work

* FoM values are obtained using Eq. 2 with the smallest three data points of nonzero values shown in Fig. 4C and fig. S20.

† EPSPS, 5-enolpyruvylshikimate 3-phosphate synthase.

‡ Digital integration time, 0.25 s.

§ Digital integration time, 10 s.

## MATERIALS AND METHODS

### Device fabrication

The fabrication of spiral Au metastructures was carried out by irradiating K_2_PdCl_6_ solution (5 mM) on Au films (100 nm; thermally evaporated at the rate of 0.1 nm/s) with a spiral vector beam. The vector beam was generated by directing a 457-nm CW laser through a half-wave plate (AHWP10-VIS, LBTEK) and a vortex phase retarder (VR1-457, LBTEK). The spiral (RH/LH) vector light can be achieved when the angle between the half-wave plate and vortex phase retarder is aligned at ±45°. These beams were subsequently focused onto Au-coated silicon substrates through a 100× dark-field (DF) objective lens [numerical aperture (NA) = 0.8; LMPLFLN100XBD, Olympus]. During the irradiation process, the Au substrate was kept in the precursor solution. The abovementioned irradiation process is based on a home-built DF microscope platform (BX53M, Olympus) as depicted in fig. S22. The laser intensity was maintained at 6.5 mW, while the irradiation duration was modulated to control the height of the spiral structures. To fabricate achiral metastructure as a control, the spiral vector beam was changed into radial vector beam by adjusting the angle of half-wave plate and the vortex phase retarder with 0° alignment while maintaining other condition the same. The obtained plasmonic metastructures were further sputter-coated with Au and characterized via scanning electron microscopy (SEM) (TESCAN MAIA3).

### Linear optical characterization and chiral sensing

The scattering and PL spectra of the spiral Au metastructures were collected through the same home-built optical setup (fig. S22). For the chiral scattering spectra measurements, white light from Halogen lamp was filtered through a linear polarizer (WP25M-UB, Thorlabs), followed by a quarter-wave plate (AQWP20-VIS, LBTEK) oriented at ±45° to generate LCP/RCP light. This circularly polarized light was then focused onto the spiral Au metastructures using a 50× DF objective lens (NA = 0.5; Olympus). Scattered light from the spiral metastructures was collected using a 50-μm optical fiber through the same DF objective, which connects to a spectrometer (QE Pro, Ocean Optics). The CDS can be obtained with the formula CDS = *S*_LCP_ − *S*_RCP_, and the *g*_CDS_ is defined as normalized CDS

$$g_{\text{CDS}}=\frac{2\text{CDS}}{S_{\text{LCP}}\left(\lambda \right)+S_{\text{RCP}}\left(\lambda \right)}$$ (7)

where *S*_LCP_ and *S*_RCP_ are the scattering intensities under LCP and RCP incidences, respectively.

For the PL measurement, the chiral PL spectra of the spiral metastructures were also collected through a 50× DF objective lens (NA = 0.5; Olympus), while a 457-nm linearly polarized CW laser with a power of 150 μW was directed to the surface of the spiral Au metastructures. The CPL emission was collected through a 500-nm long-pass edge filter (FELH0500, Thorlabs) and circular polarizers (RCP20/LCP20-VIS, LBTEK) positioned in front of the spectrometer. The CDE is defined as *I*_LCP_ − *I*_RCP_, while the *g*_lum_ is calculated on the basis of the following equation

$$g_{\text{lum}}=\frac{2\text{CDE}}{I_{\text{LCP}}\left(\lambda \right)+I_{\text{RCP}}\left(\lambda \right)}$$ (8)

where *I*_LCP_ and *I*_RCP_ represent the PL signals measured using LCP and RCP circular polarizers, respectively.

For linear chiral sensing, the CDS spectra of LH and RH spiral Au metastructures were first obtained, and then the entire substrate was immersed in BSA aqueous solution (10^−6^ to 1 mM) for 40 min, followed by gently rinsing with deionized water and blow drying. The CDS spectra of LH and RH spiral Au metastructures with adsorbed BSA were acquired again, from which we can get the relative peak shifts referred as Δλ_L_ and Δλ_R_. Last, the calibration curve of the spectral asymmetry factor ΔΔλ = Δλ_L_ − Δλ_R_ at different BSA concentrations can be obtained.

### Nonlinear optical characterization and chiral sensing

For the SHG and THG measurement, a femtosecond laser (PHAROS, Light Conversion) was used as the fundamental excitation light source ranging from 1180 to 1280 nm with a variable power of 1.0 to 5.2 mW. The laser beam is focused on the surface of the sample through a 40× reflective objective lens (NA = 0.5; Thorlabs), with a spot diameter of ∼2 μM. The SHG and THG signals were collected by using a 700-nm short-pass edge filter (MEFH10-700SP, LBTEK) placed before the spectrometer (SpectraPro HRS-500, Princeton Instruments). The digital integration time for all the nonlinear spectra is 10 s. All the SHG and THG spectra were recorded at room temperature. We obtain the nonlinear conversion efficiency (η) of SHG and THG following the definition

$$\eta_{\text{SHG},\text{THG}}=\frac{I_{2\omega ,3\omega }}{I_{\omega }}$$ (9)

where $I_{\omega }$, $I_{2\omega }$, and $I_{3\omega }$ are FW, SHG, and THG intensities, respectively.

The chiral SHG and THG spectra were detected with circular polarizers (RCP20/LCP20-VIS, LBTEK) installed in front of the spectrometer. A flat gold film was used as a blank reference to exclude any potential artifact of polarization in the reflected optical path (fig. S23). The CP_SHG,THG_ is defined as $I_{\text{LCP}}^{2\omega ,3\omega }\left(\lambda \right)-I_{\text{RCP}}^{2\omega ,3\omega }\left(\lambda \right)$, and the *g*_SHG,THG_ is calculated based on the following equation

$$g_{\text{SHG},\text{THG}}=\frac{2\left[I_{\text{LCP}}^{2\omega ,3\omega }\left(\lambda \right)-I_{\text{RCP}}^{2\omega ,3\omega }\left(\lambda \right)\right]}{I_{\text{LCP}}^{2\omega ,3\omega }\left(\lambda \right)+I_{\text{RCP}}^{2\omega ,3\omega }\left(\lambda \right)}$$ (10)

where ω is the frequency of the FW, $I_{\text{LCP}}^{2\omega ,3\omega }\left(\lambda \right)$ and $I_{\text{RCP}}^{2\omega ,3\omega }\left(\lambda \right)$ are the SHG and THG signals detected with LCP and RCP circular polarizers, respectively.

For the CP_SHG_ measurement of chiral molecules, the CP_SHG_ spectra of the spiral and achiral Au metastructures immersed in water were firstly recorded as the background CP_SHG_ (CP_SHG_bkg_). Then, the aqueous solutions containing d-PA, l-PA, and their racemic mixtures (d-/l-PA) at varying concentrations were dropped on the same samples for CP_SHG_hybrid_ measurement. During signal acquisition, we started collecting the nonlinear optical signals after the laser had been turned on for more than 20 s, when the spectra were stable. After each measurement, the spiral Au metastructures were soaked in ethanol for 30 min and subsequently rinsed thoroughly with DI water to remove adsorbed chiral molecules. After the measurement completes, we obtain the net CP of the chiral molecules with the formula

$$∆{\text{CP}}_{\text{SHG}}={\text{CP}}_{\text{SHG}\_\text{hybrid}}-{\text{CP}}_{\text{SHG}\_\text{bkg}}$$ (11)

For the nonlinear chiral sensing of BSA proteins, the CP_SHG_bkg_ of LH spiral Au metastructures was firstly obtained. Then, the whole substrate was immersed in the aqueous solution of BSA (0.1 to 100 nM) for 40 min, followed by gently rinsing with DI water and blow drying. The same procedure was performed to collect the CP_SHG_hybrid_ spectra except the measurements were done in air. Last, we can reach the ΔCP_SHG_ spectra of BSA with Eq. 11.

In the abovementioned sensing measurement, the CP_SHG_ (∼20) of achiral metastructure is used as the noise level. The LoD of BSA sensing is the averaged value based on three sets of measurements with different samples.

### Simulations

Finite element method was used to numerically simulate the SHG spectra and the near field profiles of three-dimensional spiral Au metastructures. The refractive index of Au was adopted from the Johnson-Christy data, and a perfectly matched layer was used as the boundary condition. We constructed a spiral metastructure with a diameter of 1.2 μm and a height of 1 μm on an Au film substrate. The near *E*-field and optical chirality are obtained using equations

$$\nabla \times \left(\nabla \times E\right)-k_{0}^{2}\varepsilon_{r}E=0$$ (12)

B=(∇×E)/iω (13)

$$C=\frac{-\varepsilon_{0}}{2\omega }\text{Im}\left(E^{*}\cdot B\right)$$ (14)

The SHG signals are obtained by theoretically approximating the surface nonlinear polarization current of Au and the weak contribution of bulk nonlinear polarization using following equation (*71*).

$$P_{\text{surf},\;⊥}\left(r,2\omega \right)=\chi_{⊥⊥⊥}E_{⊥}\left(r,\omega \right)E_{⊥}\left(r,\omega \right)$$ (15)

$$P_{\text{surf},∥}\left(r,2\omega \right)=\chi_{∥∥⊥}E_{∥}\left(r,\omega \right)E_{⊥}\left(r,\omega \right)$$ (16)

$$P_{\text{bulk}}\left(r,2\omega \right)=\gamma_{\text{bulk}}\nabla \cdot \left[E\left(r,\omega \right)\cdot E(r,\omega )\right]$$ (17)

where $\chi_{\text{surf}}$ and $\gamma_{\text{bulk}}$ are the second-order surface susceptibility tensor and the bulk susceptibility of materials, respectively. ⊥ and ∥ represent perpendicular and parallel to the surface. The associated surface current and bulk current are given by $j_{\text{surf}}=\partial P_{\text{surf}}/\partial t$ and $j_{\text{bulk}}=\partial P_{\text{bulk}}/\partial t$, respectively.

THG signals are calculated on the basis of a third harmonic contribution term to replace the surface current contribution, while other parts are consistent with SHG settings. The LCP and RCP signals of SHG/THG are calculated with circularly polarized collection by dividing the electric field vector into $E_{\theta }-iE_{\varphi }$ and $E_{\theta }+iE_{\varphi }$ in the far-field domain when integrating the scattering cross section, respectively.

## Supplementary Materials

### This PDF file includes:

Supplementary Text S1 to S9 Figs. S1 to S23 References
