# Supplementary material for: Ultrasensitive chiral detection by nonlinear chiroptics in spiral plasmonic metastructures surpasses linear limits
Source: Sci Adv. 2026 Sep 23;12(39):eaed9129. doi: 10.1126/sciadv.aed9129 (PMC13600241; doi:10.1126/sciadv.aed9129)
Supplement: Supplementary file 1 [file sciadv.aed9129_sm.pdf]

Science Advances
AAAS

Supplementary Materials for

Ultrasensitive chiral detection by nonlinear chiroptics in spiral plasmonic metastructures surpasses linear limits

Yong Tan *et al.*

Corresponding author: Tao Ding, t.ding@whu.edu.cn

Sci. Adv. **12**, eaed9129 (2026)
DOI: 10.1126/sciadv.aed9129

This PDF file includes:

Supplementary Text S1 to S9
Figs. S1 to S23
References

Supplementary Text

1. Fabrication of Spiral Au Metastructures and their Optical characterizations

For the laser direct writing of spiral metastructures, we first submerged a smooth and dense Au film in an aqueous solution of K_2PdCl_6 and then placed it under an optical microscope. A 457-nm continuous wave laser with linear polarization was converted into a spiral vector beam, which was then focused on the Au films through a 100× objective (Fig. S22). The excited hot carriers of Au by the laser beam facilitate the following photo-oxidative etching of Au (Eq. 1)(*73*), which can then be reduced back to Au (0) under photolysis (Eq. 2)(*74*).

$$2\mathrm{Au(s)} + \mathrm{PdCl_6^{2-}} + \mathrm{hv} \rightarrow 2[\mathrm{AuCl_2}]^- + \mathrm{Pd^{2+}} + 2\mathrm{Cl^-} \quad (1)$$

$$[\mathrm{AuCl_2}]^- + \mathrm{hv} \rightarrow \mathrm{Au(s)} + \mathrm{Cl^-} + \mathrm{Cl^{\cdot}} \quad (2)$$

As the photochemical etching can result in a roughened Au surface, the photoreduction growth of Au in the later stage preferentially takes place along the polarization of the spiral vector beam due to the locally enhanced light field of plasmons(*20*, *68*).

The generated spiral Au enantiomers (insets of Fig. S1) show opposite responses in the scattering of LCP and RCP incidences (Fig. S1A, B). Similarly, PL from these spiral enantiomers also exhibits opposite intensities of LCP and RCP emission (Fig. S1C, D).

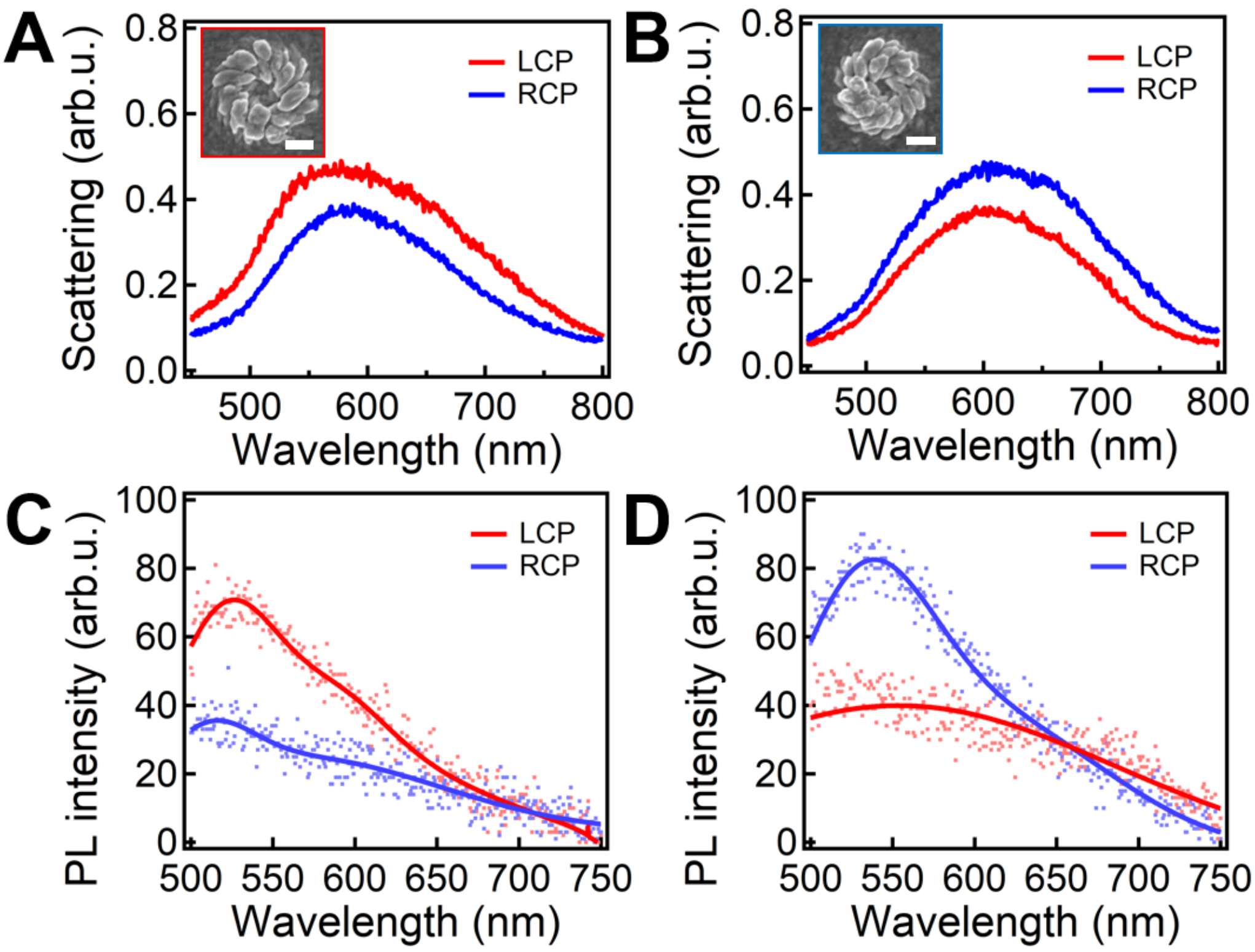

Fig. S1. Linear chiroptic properties of spiral metastructures. Chiral scattering spectra of (A) LH and (B) RH spiral Au metastructures Insets are the corresponding SEM images of spiral Au metastructures. Scale bars are 500 nm. CPL spectra of (C) LH and (D) RH spiral Au metastructures.

The dark field scattering spectrum of spiral Au metastructures exhibit plasmonic resonances around the fundamental wavelength (Fig. S2), which is crucial for the enhancement of SHG and THG processes.

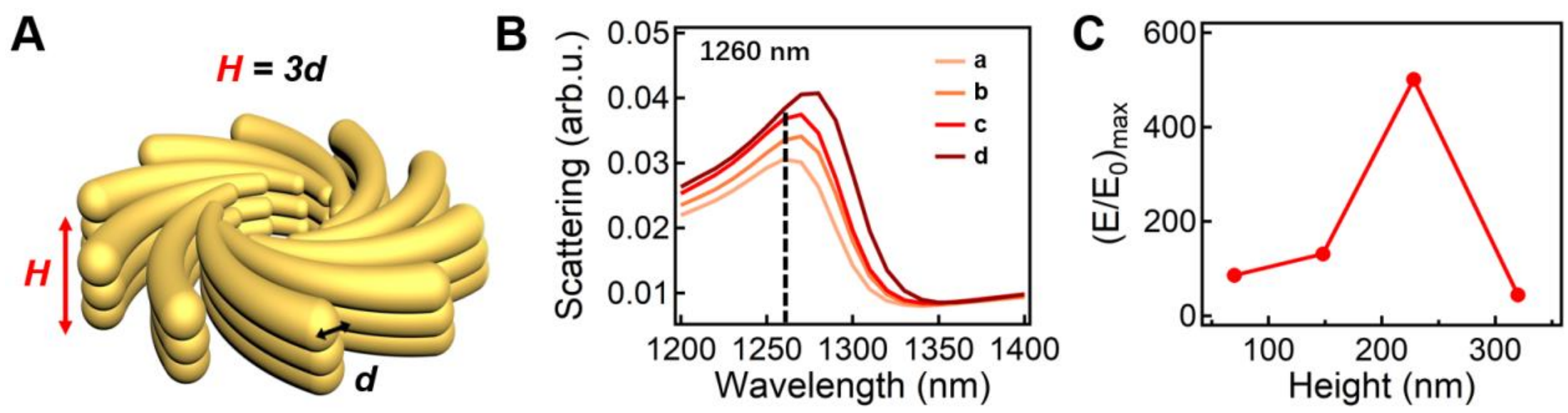

Fig. S2. Simulations of spiral Au metastructure with different height. (A) Simulation model of spiral arm with defined parameters; (B) Dark field scattering spectra of spiral Au metastructures with different heights; (C) Change of E-field enhancement of spiral Au metastructures with different heights. a) H = 72 nm; b) H = 148 nm; c) H = 228 nm; d) H = 320 nm.

For the achiral Au metastructure, a radially polarized vector beam was applied when the angle between the laser polarization and the fast axis of the vortex plate was aligned in 0°. The generated achiral Au metastructure (inset of Fig. S3) shows negligible difference of LCP and RCP signals of SHG (Fig. S3A), which exhibits no CP_{SHG} response (Fig. S3B).

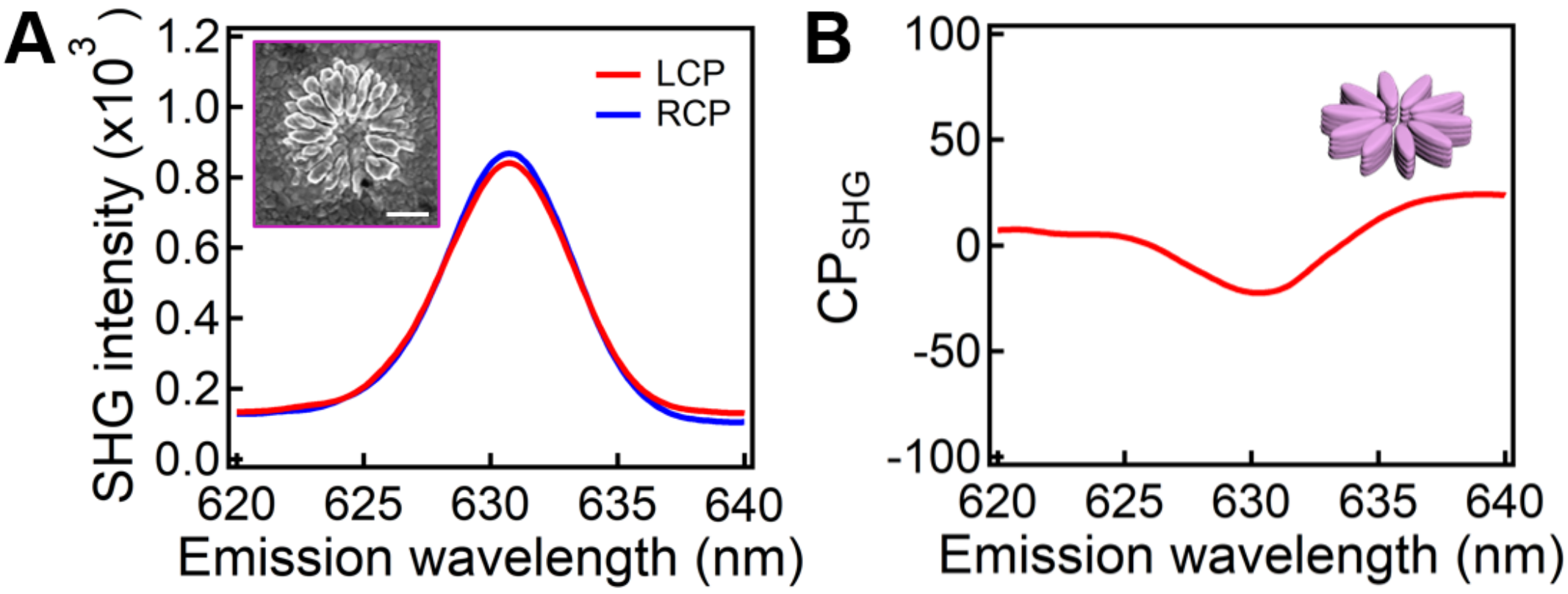

Fig. S3. SHG spectra of achiral Au metastructure. (A) SHG spectra of achiral metastructure detected with LCP and RCP waveplates. The fundamental wavelength of excitation is 1260 nm with a power of 4.0 mW. Inset is the SEM image of the achiral Au metastructure. Scale bar is 500 nm. (B) CP_{SHG} spectrum of the achiral Au metastructure.

2. Numerical Simulation of Superchiral Field

The superchiral field distribution around the spiral metastructure was calculated using the finite element method. Specifically, a circularly polarized plane wave was applied with normal incidence on the spiral Au metastructure to calculate the electric and magnetic field enhancement. The optical chirality (C) of the spiral Au metastructures is calculated based on the simplfied equation(*75*)

$$C = \frac{-\varepsilon_0}{2\omega}\mathrm{Im}(\mathbf{E}^* \cdot \mathbf{B}) \quad (3)$$

where **E** and **B** are the electric and magnetic fields of the spiral metastructures, respectively. The value of C is normalizd to the optical chirality of the light field without any structures (C_0) to show the optical chirality enhancement ($C/|C_0|$). Here C_0 is defined as

$$C_0 = \frac{-\varepsilon_0}{2\omega}\mathrm{Im}(\mathbf{E}_0^* \cdot \mathbf{B}_0) \quad (4)$$

The optical chirality enhancement of the spiral Au metastructure under LCP and RCP incidences can induce an optical chirality difference ($\Delta \mathrm{C} = C_{\mathrm{LCP}} - C_{\mathrm{RCP}}$), which has a critical influence on the chiroptic properties(*76*). Such a difference at the second (2ω) and third (3ω) harmonic frequencies show opposite signs (Fig. S4). Specifically, at the THG wavelength (420 nm), the superchiral field $\Delta C/|C_0|$ is dominated by the negative values (blue colors in Fig. S4A), while at the SHG wavelength (630 nm), the superchiral field $\Delta C/|C_0|$ shows a positive background (red colors in Fig. S4B). As such, the chiroptic response at these wavelengths of THG and SHG leads to the negative and positive values of g_{THG} and g_{SHG}, respectively.

Note that at the fundamental wavelength (1260 nm), the optical chirality difference is relatively weak as compared to the harmonic wavelengths (Fig. S4C), therefore the large g-factor is mainly contributed by the chiral scattering process at the harmonic frequencies.

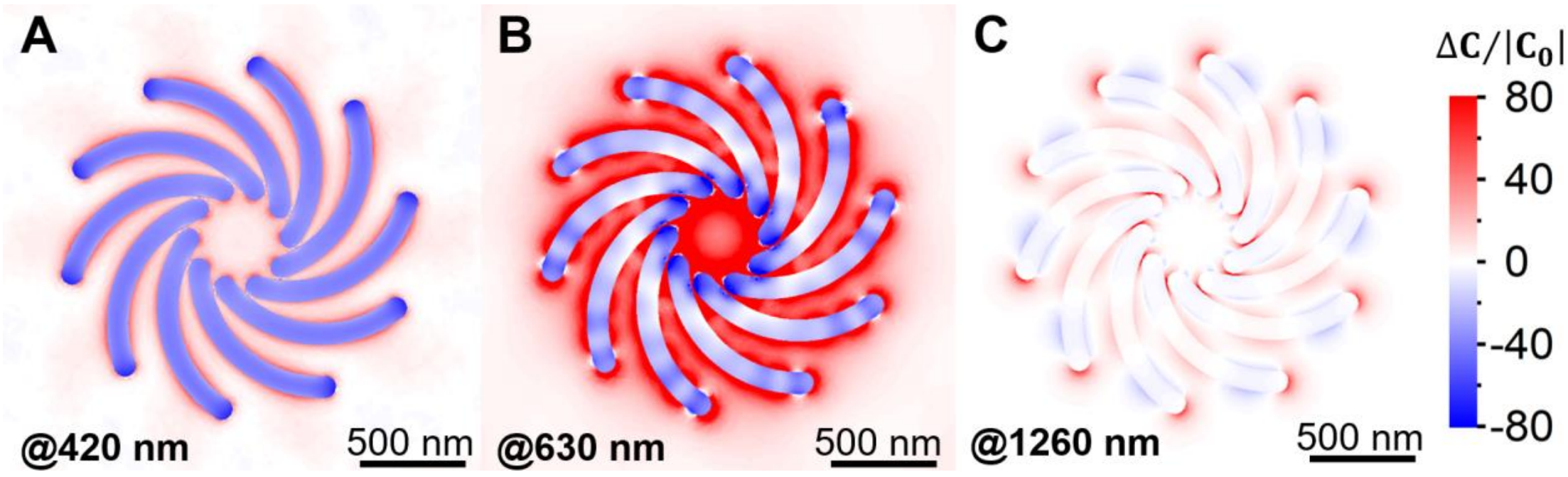

Fig. S4. Simulated optical chirality difference ($\Delta C/|C_0|$) in the spiral Au metastructures at different wavelengths. (A) 420 nm (3ω). (B) 630 nm (2ω). (C) 1260 (ω).

3. Influence of the Height of Spiral Metastructures on Their Nonlinear Chiroptic Response

We can adjust the height of the spiral Au metastructures by adjusting the irradiation time. Fig. S5 shows the nonlinear chiroptic response of the LH spiral metastructure of at variable heights. As the height increases, the intensity of SHG and THG gradually increases. However, their difference between LCP and RCP shows a non-monotonic increase. The largest difference appears when the height reaches 0.5 μm at an irradiation time of 120 s, which exhibits g_{SHG} of ~0.7 and g_{THG} of ~-0.6 (Fig. S6). This is likely because the closed nanogaps within the spiral Au metastructure notably reduce the local E-field enhancement for nonlinear chiroptic signals (Fig. S2C). This structure was adopted as the typical sample for nonlinear chiral sensing measurement in the later section.

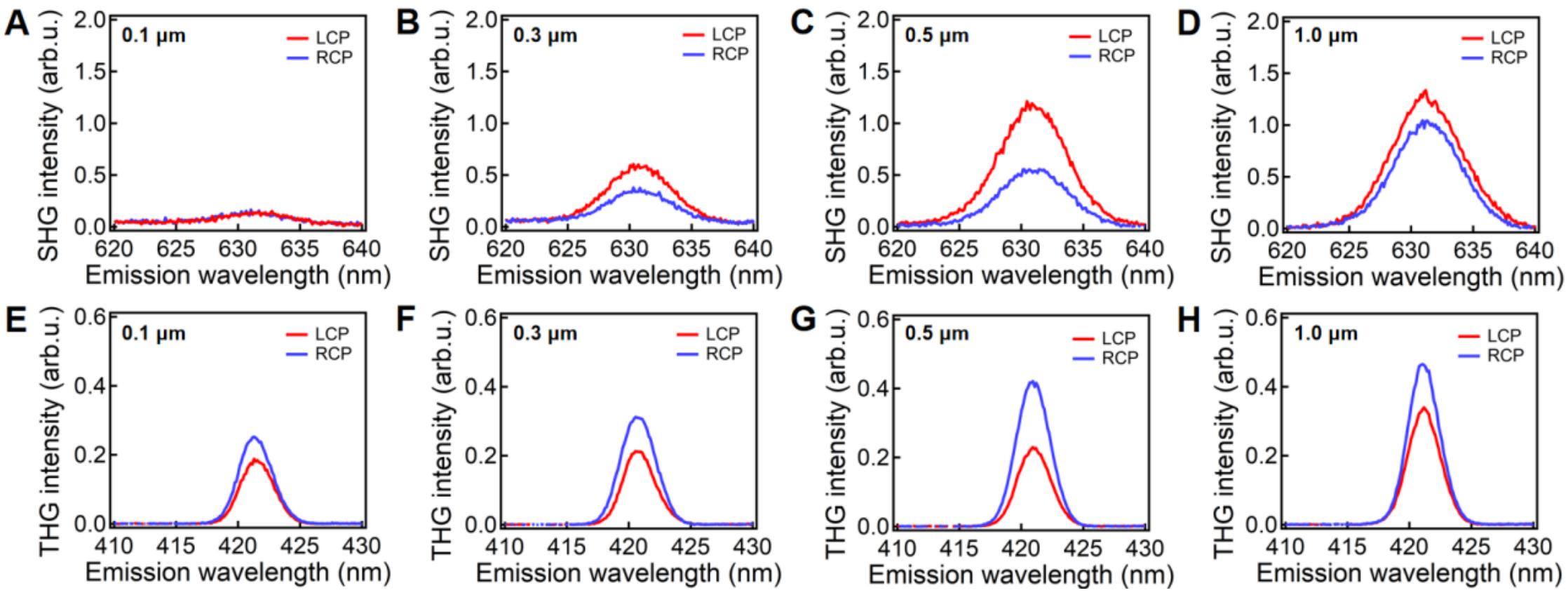

Fig. S5. Influence of the height of the spiral metastructures on the nonlinear chiroptic response. Chiral SHG spectra of LH spiral metastructures of different heights. (A) 0.1 μm, (B) 0.3 μm, (C) 0.5 μm, (D) 1.0 μm. Chiral THG spectra of LH spiral metastructures of different heights. (E) 0.1 μm, (F) 0.3 μm, (G) 0.5 μm, (H) 1.0 μm.

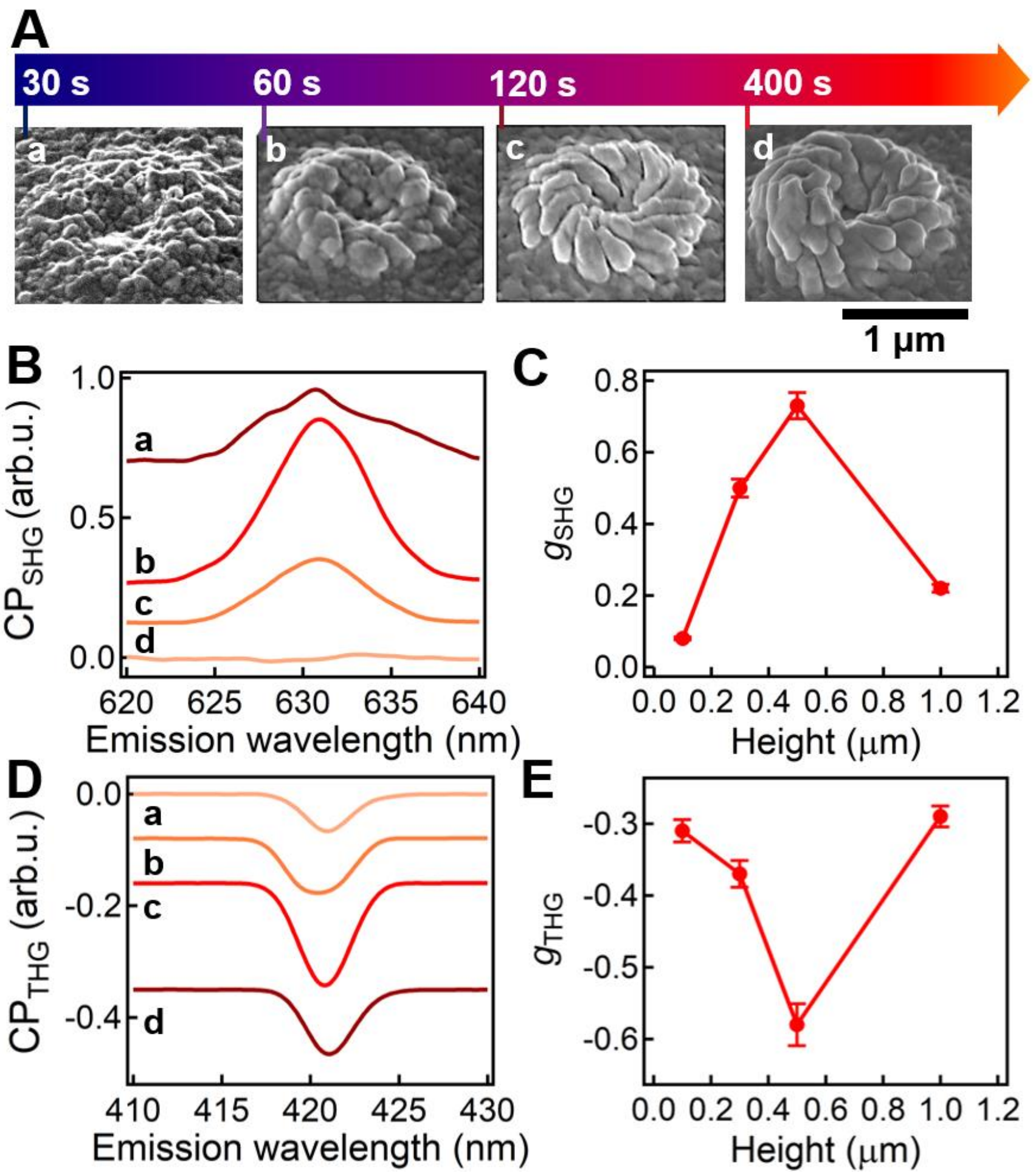

Fig. S6. Influence of structural height on the nonlinear chiroptic properties of 3D spiral metastructures. (A) Growth of 3D spiral metastructures under different laser direct writing times: a) 30 s; b) 60 s; c) 120 s; d) 400 s. Measured (B) CP_{SHG} and (D) CP_{THG} spectra of spiral metastructures at different height, and the change of their (C) g_{SHG} and (E) g_{THG} with the height of spiral metastructures.

4. Nonlinear Chiroptic Sensing of Small Chiral Molecules Based on Spiral Au Metastructures

As the spiral Au metastructures are intrinsically chiral, we need to first measure the CP_{SHG} spectra of the LH and RH spiral Au metastructures as a background signal (baseline) for nonlinear chiroptic sensing measurement. Specifically, we measured the CP_{SHG} of spiral Au metastructures with the presence of pure water (dashed lines in Fig. S7 and 8) as the CP_{SHG_bkg}. And then, we measured the CP_{SHG} of the same spiral Au metastructures with the presence of D-PA and L-PA aqueous solutions with increasing concentration (solid lines in Fig. S7 and 8) as the CP_{SHG_hybrid}. After each measurement, the spiral Au metastructures were immersed and rinsed in ethanol for 30 minutes to remove the residual chiral molecules on the surface. Finally, we subtracted the background CP_{SHG} to obtain the ΔCP_{SHG} spectra, which are shown in the main manuscript (Fig. 3A, B).

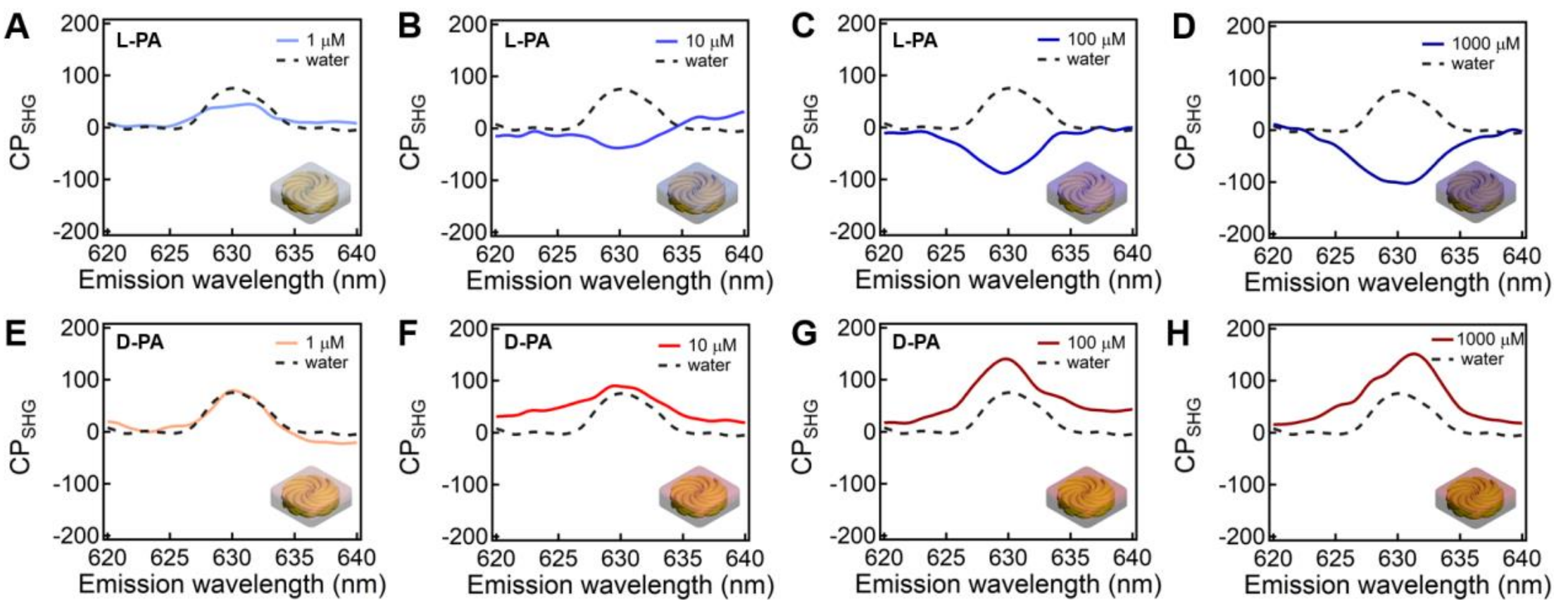

Fig. S7. Nonlinear chiroptic sensing based on LH spiral plasmonic metastructures. CP_{SHG} spectra of LH spiral plasmonic metastructures with presence of L-PA of different concentrations. (A) 1 μM, (B) 10 μM, (C) 100 μM and (D) 1000 μM. CP_{SHG} spectra of LH spiral plasmonic metastructures with the presence of D-PA of different concentrations. (E) 1 μM, (F) 10 μM, (G) 100 μM and (H) 1000 μM. The dashed lines are the CP_{SHG} spectra of LH spiral plasmonic metastructure without any chiral molecules.

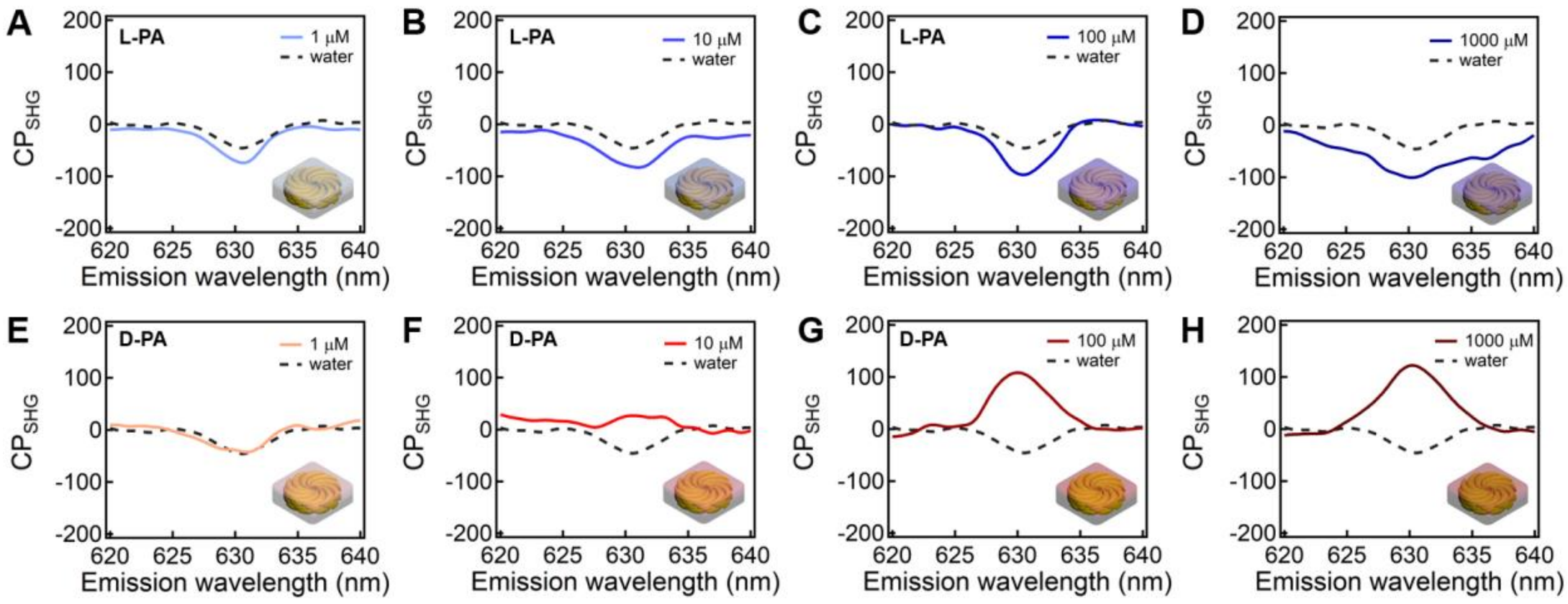

Fig. S8. Nonlinear chiroptic sensing based on RH spiral plasmonic metastructures. CP_{SHG} spectra of RH spiral plasmonic metastructures with presence of L-PA of different concentrations. (A) 1 μM, (B) 10 μM, (C) 100 μM and (D) 1000 μM. CP_{SHG} spectra of RH spiral plasmonic metastructures with the presence of D-PA of different concentrations. (E) 1 μM, (F) 10 μM, (G) 100 μM and (H) 1000 μM. The dashed lines are the CP_{SHG} spectra of RH spiral plasmonic metastructure without any chiral molecules.

We also used an achiral molecule (cysteamine) as a control to investigate its influence on the CP_{SHG} of LH and RH spiral Au metastructure. We found even with the presence of 1 mM of cysteamine, the CP_{SHG} spectra do not show notable change, which results in nearly zero ΔCP_{SHG} irrespective of whether it is a RH or LH spiral Au metastructure (Fig. S9). This control experiment further confirms the validity of the nonlinear chiroptic measurements in this work.

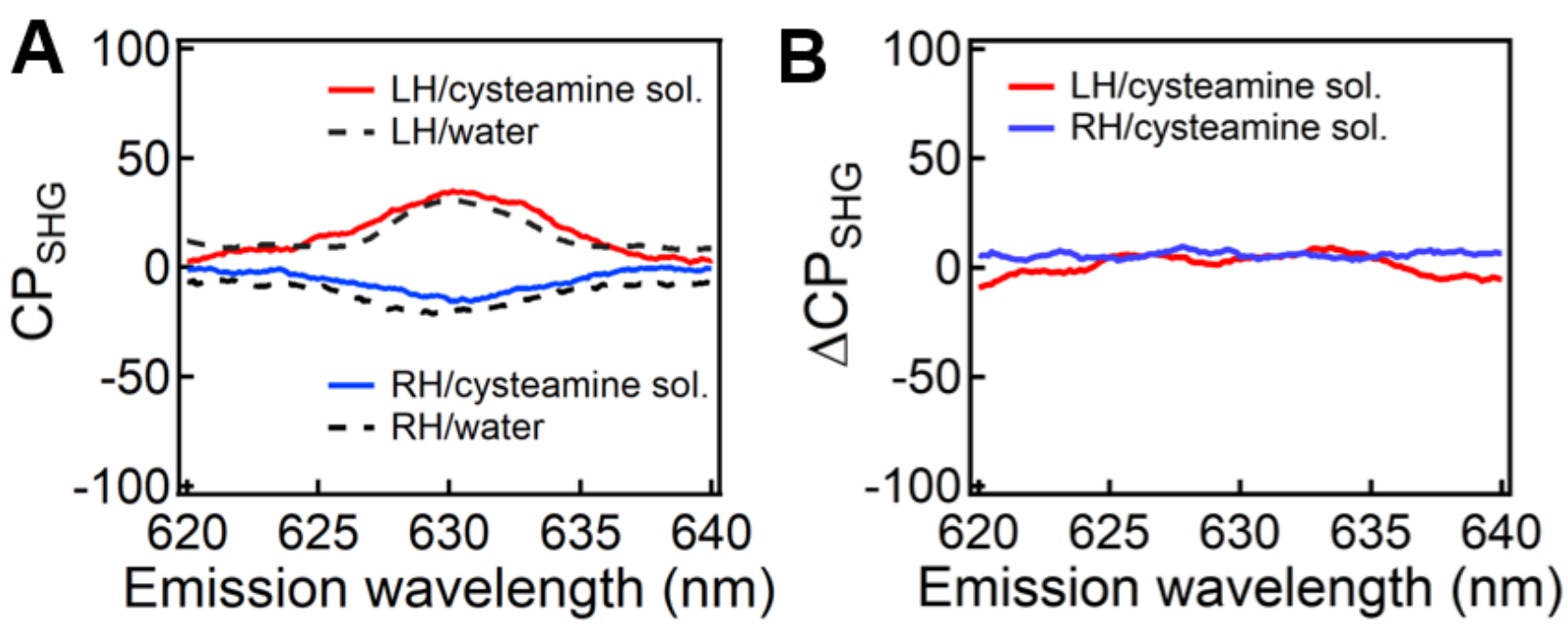

Fig. S9. Nonlinear chiroptic response of spiral Au metastructures with the presence of achiral molecules. (A) CP_{SHG} spectra of LH and RH spiral Au with presence of cysteamine (1 mM) and (B) their ΔCP_{SHG} spectra.

Under the same measurement system, we measured the THG signals of the LH and RH spiral Au metastructures with the presence of different concentrations of D-PA and L-PA molecules. A series of ΔCP_{THG} spectra at different molecular concentrations were obtained by subtracting the CP_{THG_bkg} from the CP_{THG_hybrid} (Fig. S10A, B). The calibration curves of the maximum value of ΔCP_{THG} *vs* concentration show that the THG process also exhibits a linear response to the concentration of chiral molecules, but cannot effectively distinguish the enantiomeric signals (Fig. S10C), which agrees with simulations (Fig. S10D)

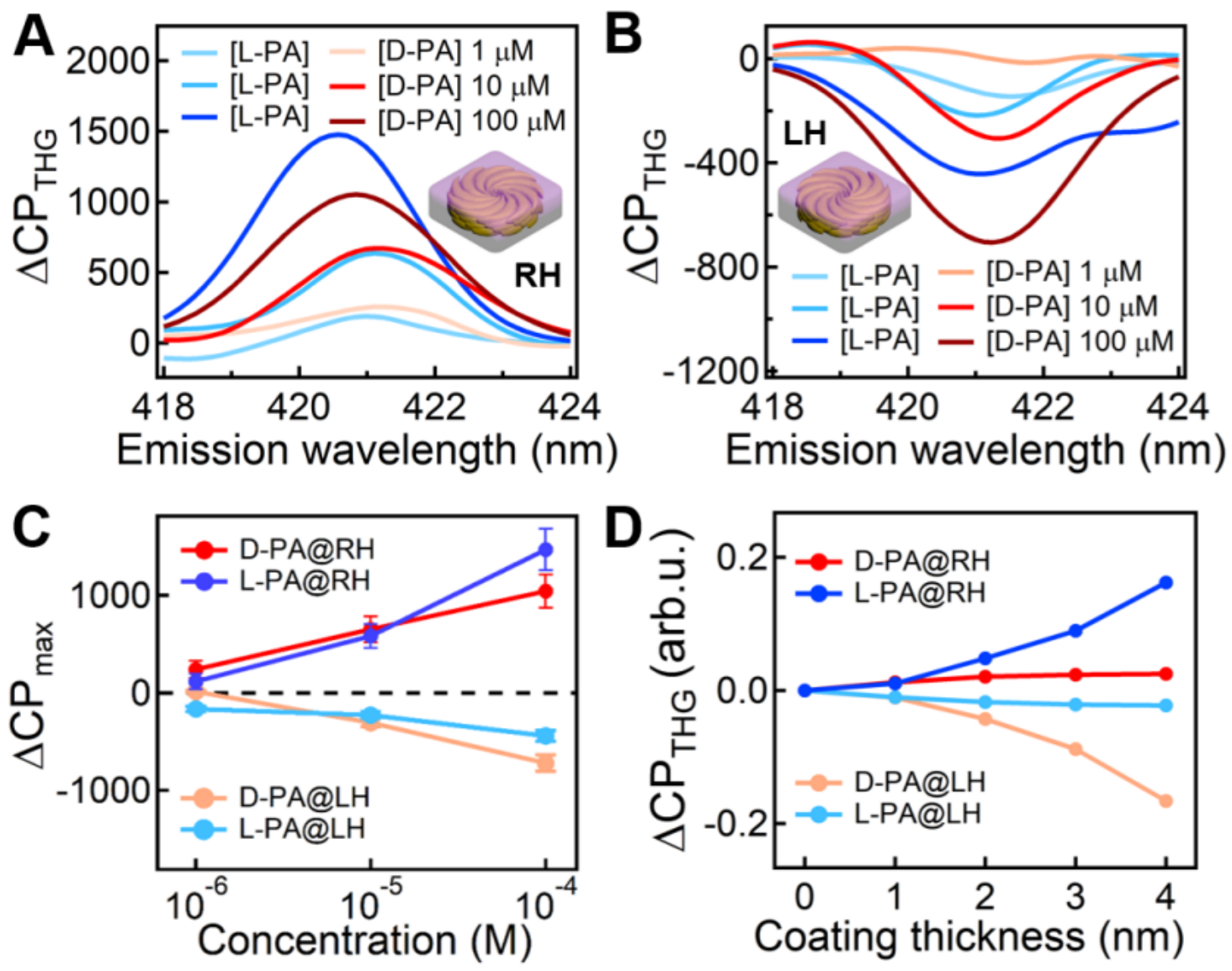

Fig. S10. THG-based chiroptic sensing with spiral plasmonic metastructures. ΔCP_{THG} spectra of (A) LH and (B) RH spiral plasmonic metastructures with presence of D-PA and L-PA solutions. (C) Change of ΔCP_{max} with concentration of D-PA and L-PA detected with RH/LH spiral Au metastructures. (D) Simulation calibration curve of ΔCP_{THG} with of D-PA and L-PA based on RH/LH spiral Au metastructures.

We also measured the nonlinear chiroptic sensing of PA with an achiral plasmonic metastructure (inset of Fig. S11). Even though the concentration of PA increases from 1 to 100 μM, no obvious increase of ΔCP_{SHG} signals can be found (Fig. S11), suggesting the important role of asymmetric E-field enhancement for the dramatic change of ΔCP_{SHG} spectra of the spiral Au metastructures with the presence of chiral molecules (Fig. 4A, B).

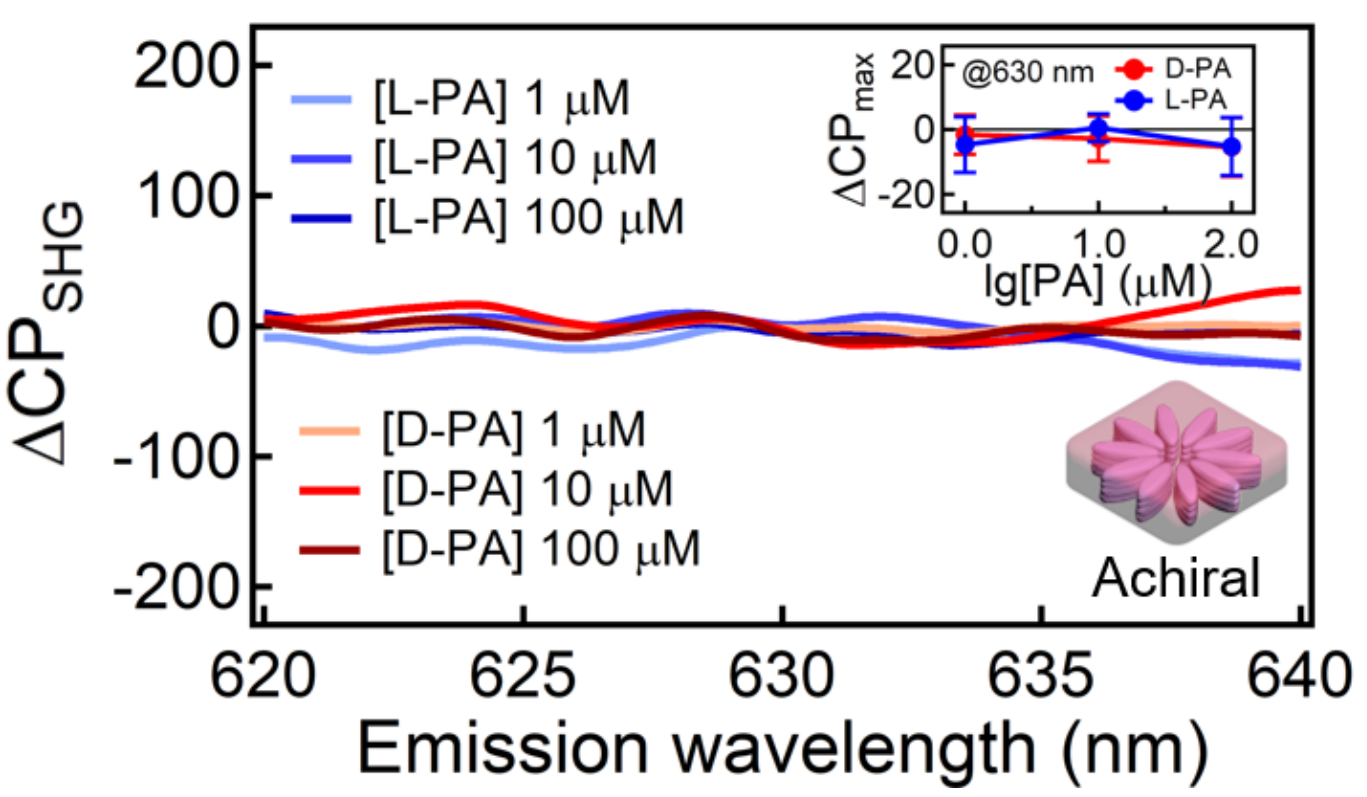

Fig. S11. ΔCP_{SHG} spectra of of an achiral Au metastructure immersed in D-PA and L-PA solutions of different concentrations. Inset is the correlation between ΔCP_{SHG} and the concentration of the enantiomers.

It is critical to measure the CP_{SHG} with the presence of amino acid that has nonspecific binding to Au. For instance, we can see a change of CP_{SHG} of the RH spiral Au metastructure with the presence of D-PA solution (Fig. S12A). When the D-PA solution was removed from the surface of the RH spiral Au metastructures right after the measurement (~10 mins) and the CP_{SHG} was measured again in air, almost no change can be found (Fig. S12B). However, if the samples were incubated with D-PA solution for a longer period of time (~40 mins) to allow sufficient surface adsorption, then we can find a dramatic change of CP_{SHG} signals (Fig. S12C), suggesting that the CP_{SHG} is highly surface sensitive, which is crucial for the monitoring of surface chemistry.

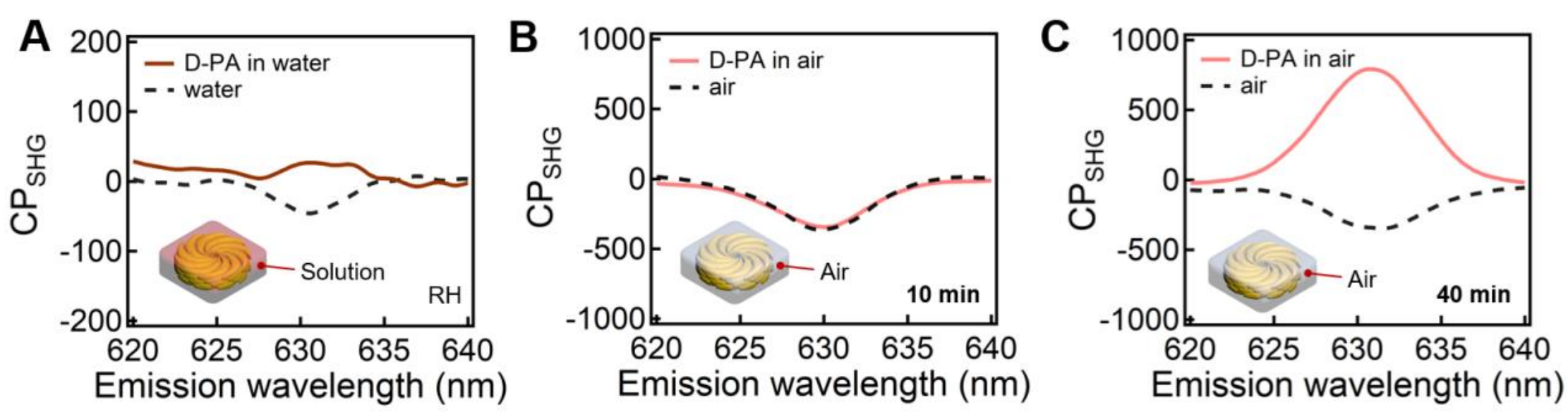

Fig. S12. Comparison of CP_{SHG} spectra for the sensing of D-PA. CP_{SHG} spectra of RH spiral plasmonic metastructures in the presence of (A) D-PA solution (10 μM) and rinsed and dried after incubation for (B) 10 mins and (C) 40 mins.

5. Linear and Nonlinear Chiroptic Sensing of Chiral Proteins Based on Spiral Au Metastructures

Many of the diseases are related to the malfunction of the proteins, which directly relates to the change of their chirality. However, diagnosis based on the detection of chirality change has been almost impossible in clinical applications due to their extremely weak chiroptic signals. Here we explore this sensing possibility with chiroptics enhanced by chiral plasmons. Specifically, we adopt Bovine Serum Albumin (BSA) as the model protein, which is adsorbed on the spiral Au metastructures via self-assembly. We ignore the orientation of the biomolecules adsorbed on the surface of the spiral Au metastructures as it is more likely to be random although it has a great impact on the ΔCP_{SHG} (Fig. S13).

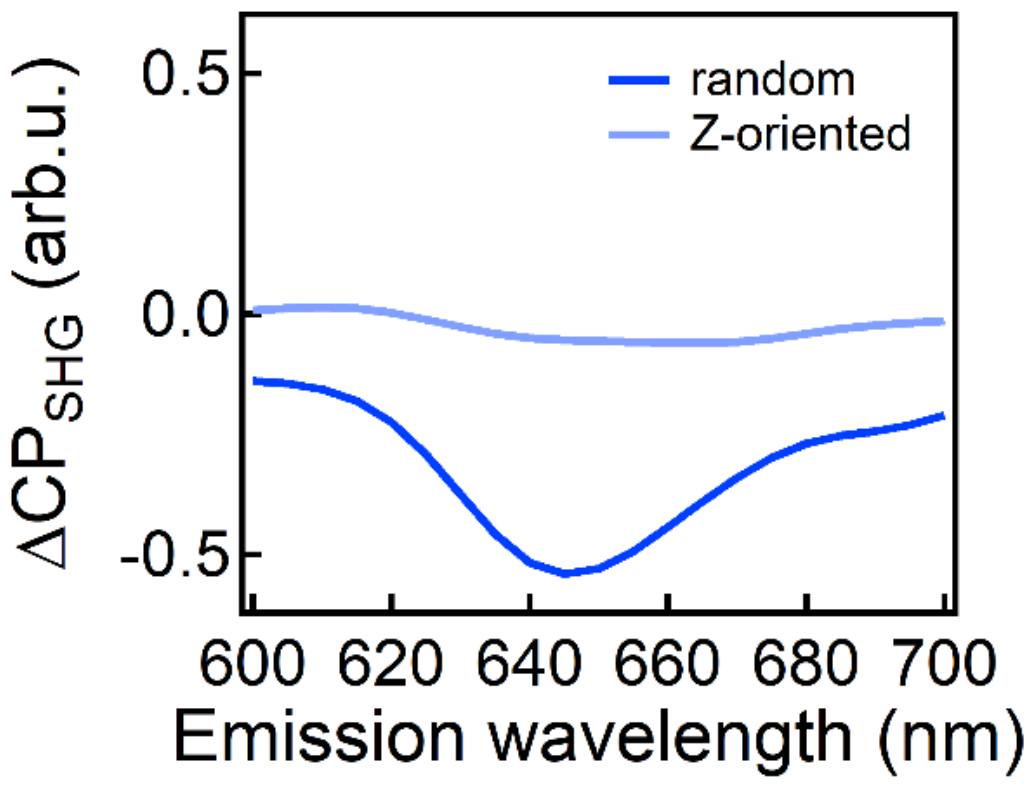

Fig. S13. Comparison of ΔCP_{SHG} calculated with chiral molecules of random and z-oriented configurations on the spiral Au metastructures.

Specifically, the BSA solutions of different concentrations were prepared and the spiral Au metastructures were soaked in the BSA solution for 30 mins, after which they were taken out and rinsed with deionized water to remove extra loosely adsorbed BSA. The samples were blow-dried for linear (CDS) and nonlinear (CP_{SHG}) chiroptic sensing measurement. To ensure the robustness of the measurement, we performed the measurement with three sets of different samples (Figs. S14-16).

Prior to nonlinear sensing measurement, the CP_{SHG} spectra of spiral metastructures were recorded as a baseline for nonlinear chiroptic sensing (dashed lines in Figs. S14-16). The CP_{SHG} spectra of spiral Au metastructures with adsorbed BSA were taken in air with the excitation of a pulsed laser beam (1260 nm, 4.0 mW).

As the concentration of BSA solution increases, the number of adsorbed BSA molecules increases proportionally so that the CP_{SHG} peak intensity gradually changes from positive to negative (solid lines in Figs. S14-16).

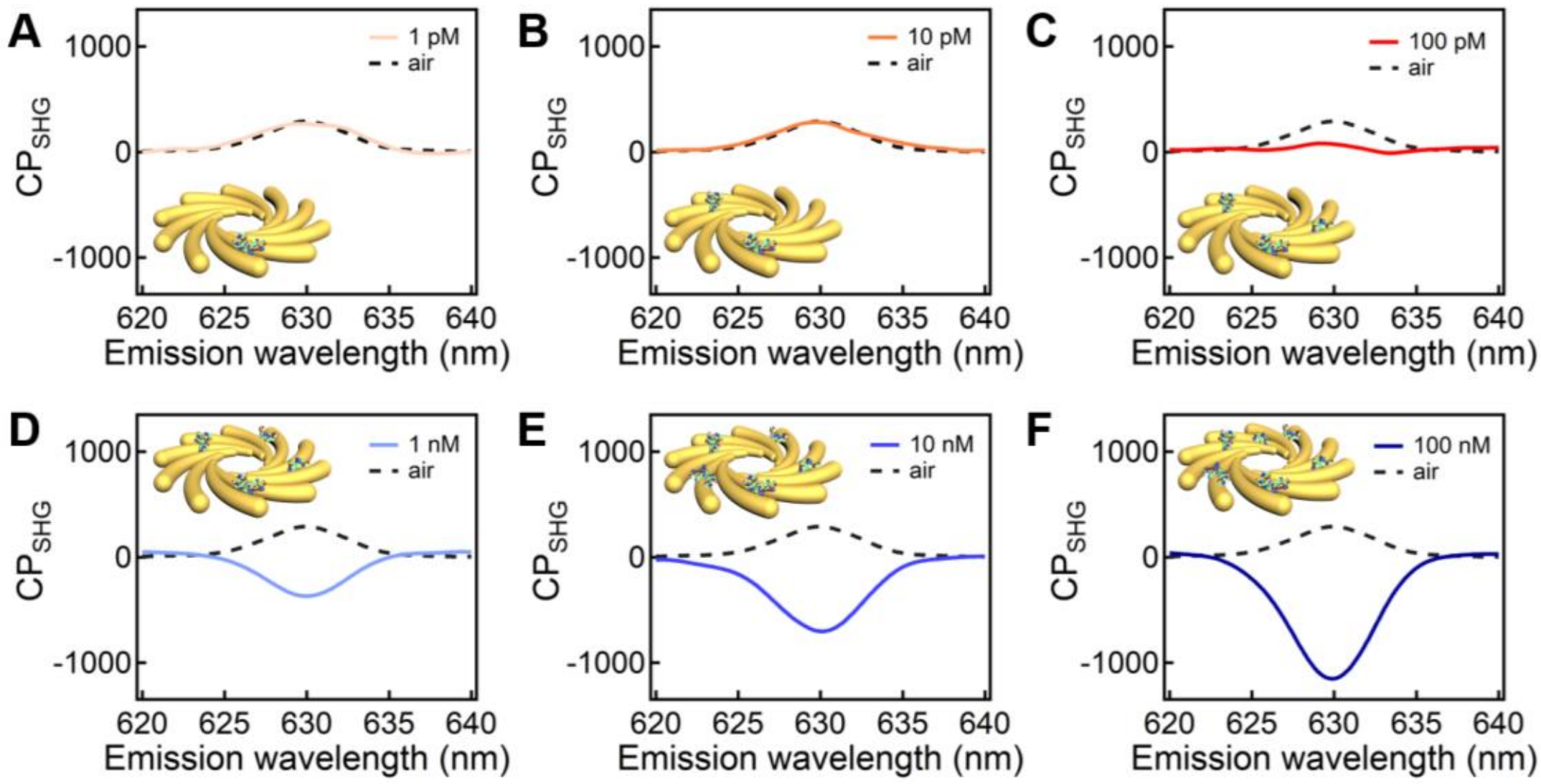

Fig. S14. **The first set of measurements for BSA proteins nonlinear chiroptic sensing based on LH spiral plasmonic metastructures.** CP_{SHG} spectra of LH spiral plasmonic metastructures coated with BSA at different concentrations. (A) 1 pM, (B) 10 pM, (C) 100 pM, (D) 1 nM, (E) 10 nM and (F) 100 nM. The dashed lines are the CP_{SHG} spectra of LH spiral plasmonic metastructure in the air. Insets are schematics of the increasing number of BSA molecules adsorbed on LH spiral Au metastructure.

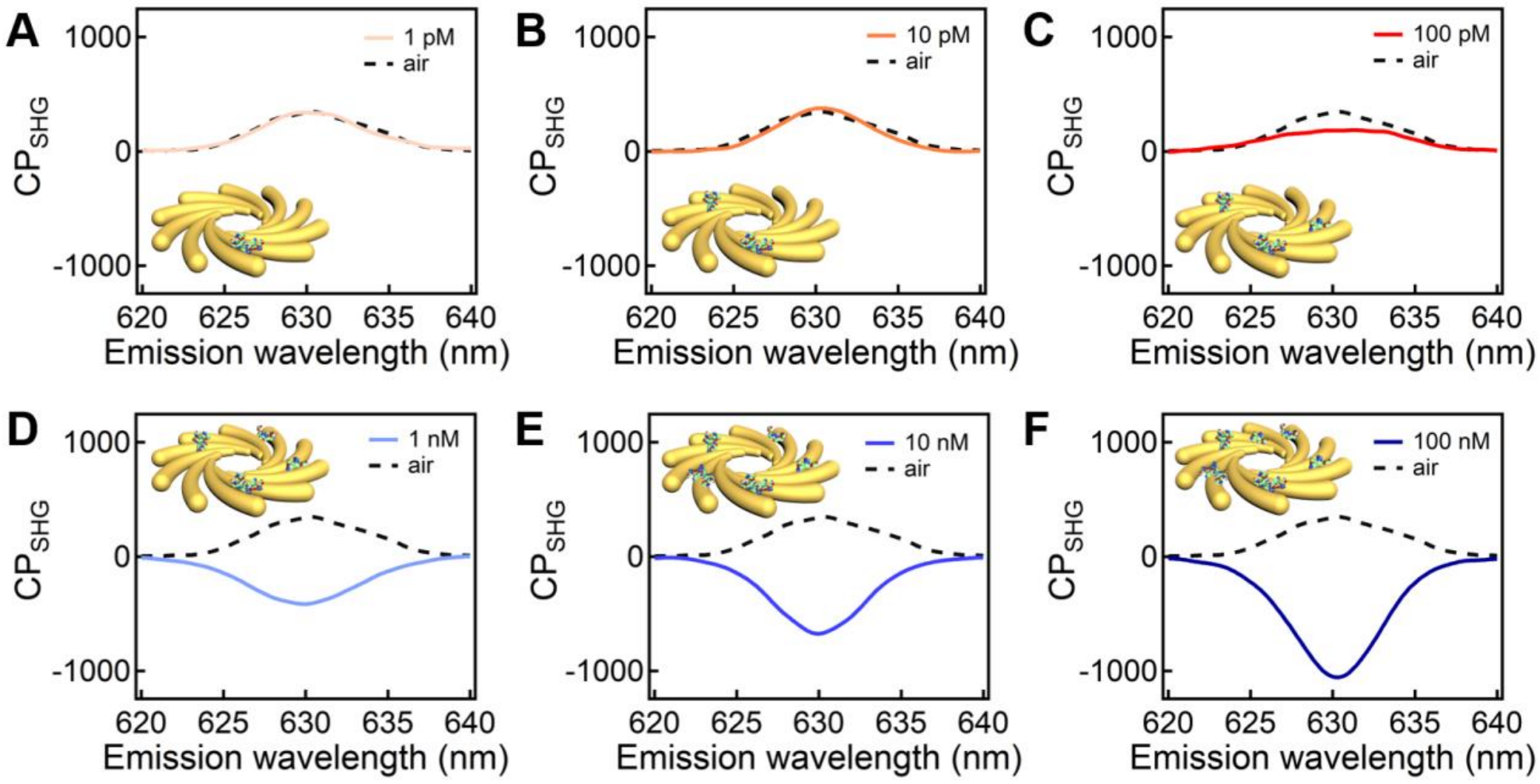

Fig. S15. **The second set of measurements for BSA proteins nonlinear chiroptic sensing based on LH spiral plasmonic metastructures.** CP_{SHG} spectra of LH spiral plasmonic metastructures coated with BSA at different concentrations. (A) 1 pM, (B) 10 pM, (C) 100 pM, (D) 1 nM, (E) 10 nM and (F) 100 nM. The dashed lines are the CP_{SHG} spectra of LH spiral plasmonic metastructure in the air. Insets are schematics of the increasing number of BSA molecules adsorbed on LH spiral Au metastructure.

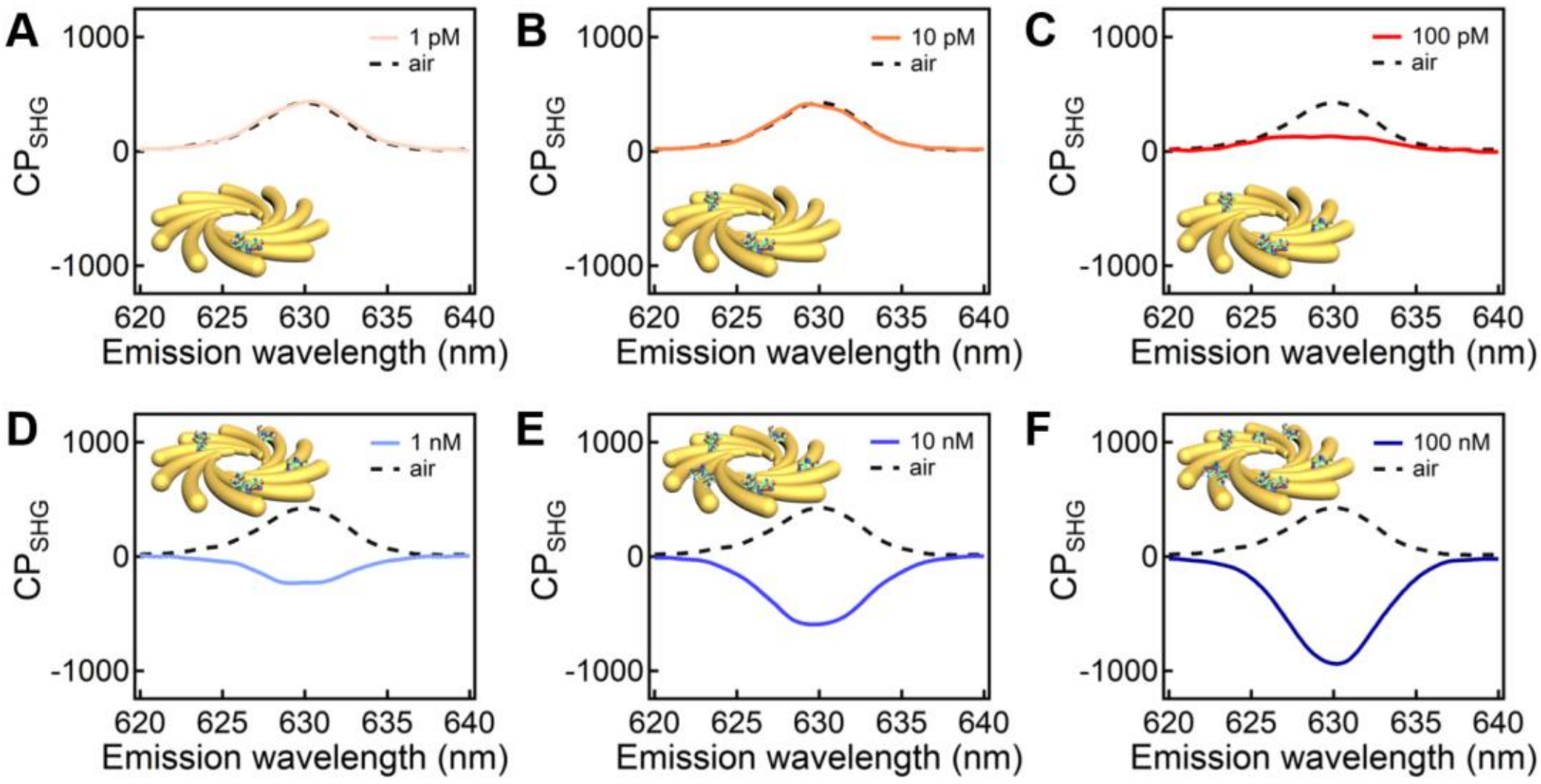

Fig. S16. **The third set of measurements for BSA proteins nonlinear chiroptic sensing based on LH spiral plasmonic metastructures.** CP_{SHG} spectra of LH spiral plasmonic metastructures coated with BSA at different concentrations. (A) 1 pM, (B) 10 pM, (C) 100 pM, (D) 1 nM, (E) 10 nM and (F) 100 nM. The dashed lines are the CP_{SHG} spectra of LH spiral plasmonic metastructure in the air. Insets are schematics of increasing number BSA adsorbed on LH spiral Au metastructure.

The relationship between the differential CP_{SHG} signal (ΔCP_{SHG}) and protein concentration (c) is described by the Temkin-II model as

$$\Delta CP_{SHG} = Aln(1 + K_0 c)$$

where K_0 is the Temkin equilibrium binding constant. The fitting of the calibration curves (Fig. S17) gives A=-174, K_0=3.7×10^{10} M^{-1} with a regression coefficient (R^2) of 0.992. Here the Temkin-II isotherm model was chosen because the Langmuir model assumes homogeneous binding sites and a single binding energy, which is inadequate for protein adsorption involving conformational changes and surface heterogeneity (*70*). As BSA is a flexible protein, and the spiral Au metasurface exhibits heterogeneous nanogaps and nanobumps that produce a distribution of binding energies, the Temkin-II model is therefore more physically appropriate for both the protein and small-molecule adsorption studied here.

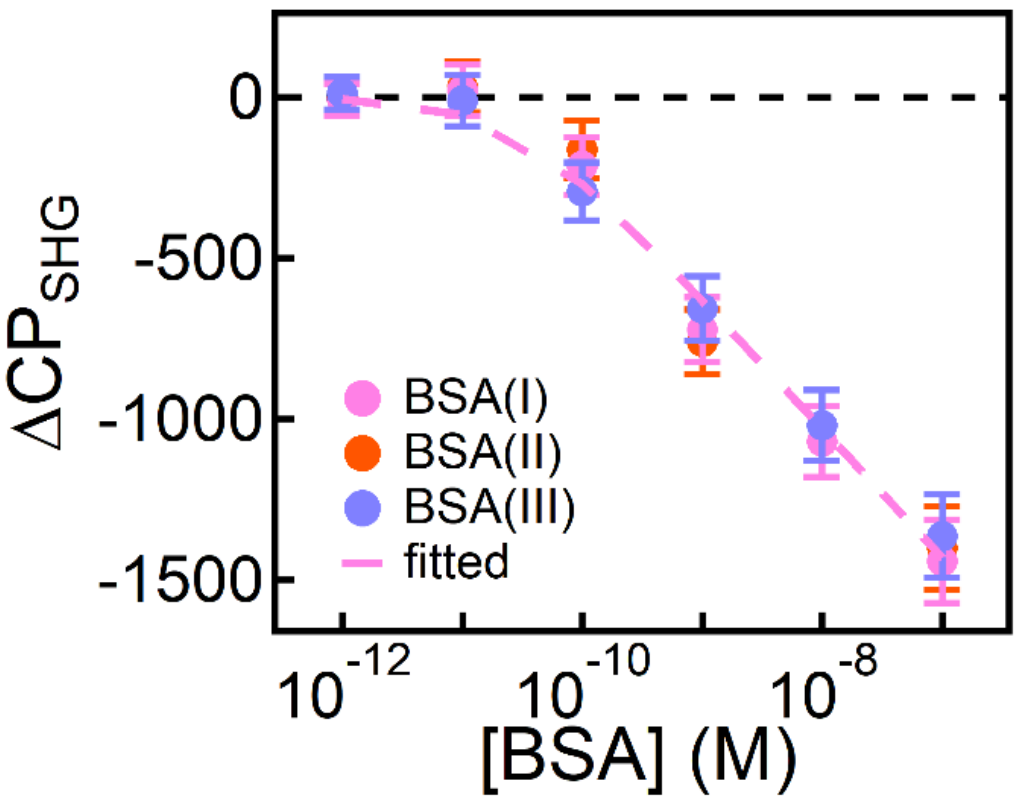

Fig. S17. **Reproducibility of the calibration.** Differential circularly polarized SHG signals (ΔCP_{SHG}) of BSA adsorption at different concentrations on spiral Au metastructures measured with three different sets of samples. (I), (II), and (III) represent the test groups. The dashed line represents the fitted result by the Temkin-II model.

The LoD was determined as the concentration corresponding to a signal equal to three times the noise level (σ=20) of the blank measurement (3σ method). Based on the above fitting equation, the calculated LoD is 11 pM.

The actual number of adsorbed BSA molecules on the spiral Au metastructure can be estimated by

$$N_{\mathrm{BSA}} = N_{\mathrm{c}} \cdot S \quad (5)$$

where N_c is the grafting density of BSA on the Au surface at a concentration of c in solution, and S is the surface area of the spiral Au metastructures. The former can be determined with a quartz crystal microbalance (QCM) while the latter can be roughly estimated by the sum of the surface areas of the inner and outer walls, as well as the top surface through the metastructure schematic diagram in Fig. S18A. Here we measured the mass change of the Au electrode with QCM after immersing it in a BSA solution of 0.1 nM (Fig. S18B). The value of $N_{0.1}$ is determined to be ~$4.4 \times 10^5/\mu m^2$.

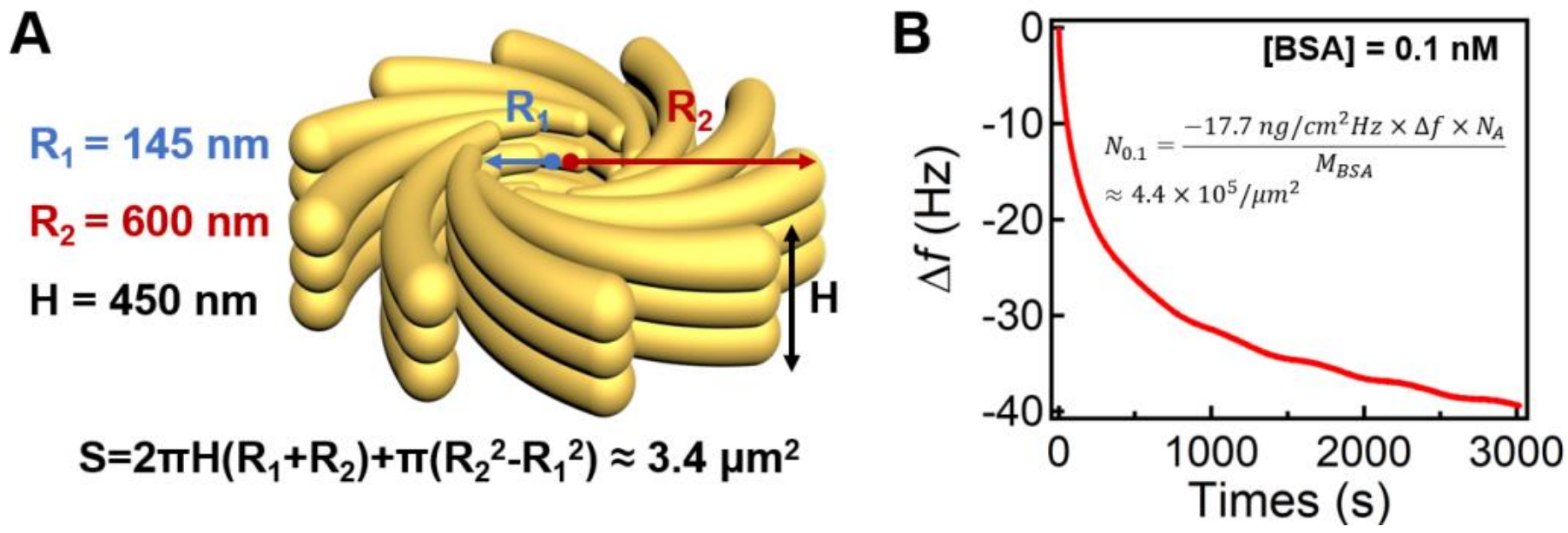

Fig. S18. Quantification of BSA molecule adsorbed on the spiral Au metastructure. (A) Schematic of spiral Au metastructure with defined parameters and surface area calculation. (B) QCM measurement of BSA grafting density on the Au surface.

The linear chiroptic sensing of BSA is based on the shift of the CDS peak ($\Delta\lambda_{L/R}$) of LH and RH spiral plasmonic metastructures, respectively (Fig. S19A, B). As the concentration of BSA decreases, the spectral dissymmetry factor ($\Delta\Delta\lambda=\Delta\lambda_L-\Delta\lambda_R$) decreases proportionally, which shows a detection limit of 1 nM (Fig. S19C).

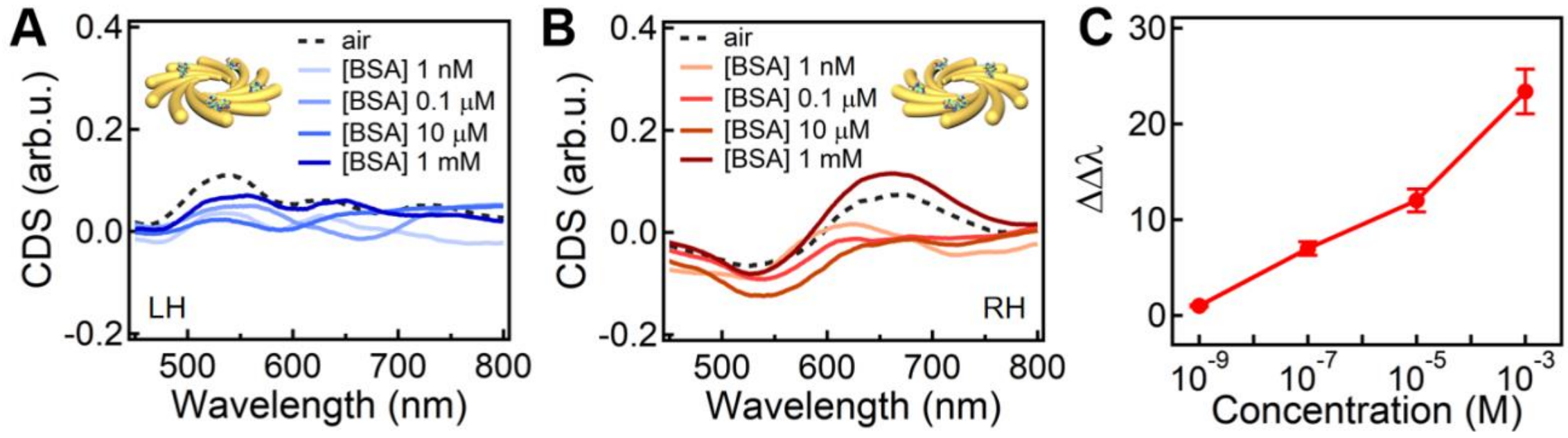

Fig. S19. Linear chiroptic sensing of L-BSA based on spiral plasmonic metastructures. CDS spectra of (A) LH and (B) RH spiral plasmonic metastructures incubated with L-BSA of different concentrations. (C) Change of $\Delta\Delta\lambda$ with BSA concentration.

6. FoM Determination of Chiral Plasmonic Sensing Devices

To quantitatively evaluate and compare the comprehensive chiral sensing performance of different chiral plasmonic sensors, we introduce a Figure of Merit (FoM) index to characterize the overall level of detection sensitivity and signal-to-noise ratio of the sensor, which is defined as:

$$\mathrm{FoM} = \frac{\Delta CD}{\Delta c \cdot \sigma} \quad (6)$$

where ΔCD refers to the changes in CD signals after the addition of chiral molecules, and Δc is the change of chiral molecule concentration. σ is the signal-to-noise ratio of the sensors which is represented by the standard deviation of the CD baselines. The data for calculating the FoM of different chiral plasmonic sensors are from the data shown in Fig. 4C, and previously published data (Fig. S20) such as the helicoid system(*23*, *26*), shuriken structures(*19*), and spiral Au racemic arrays(*27*).

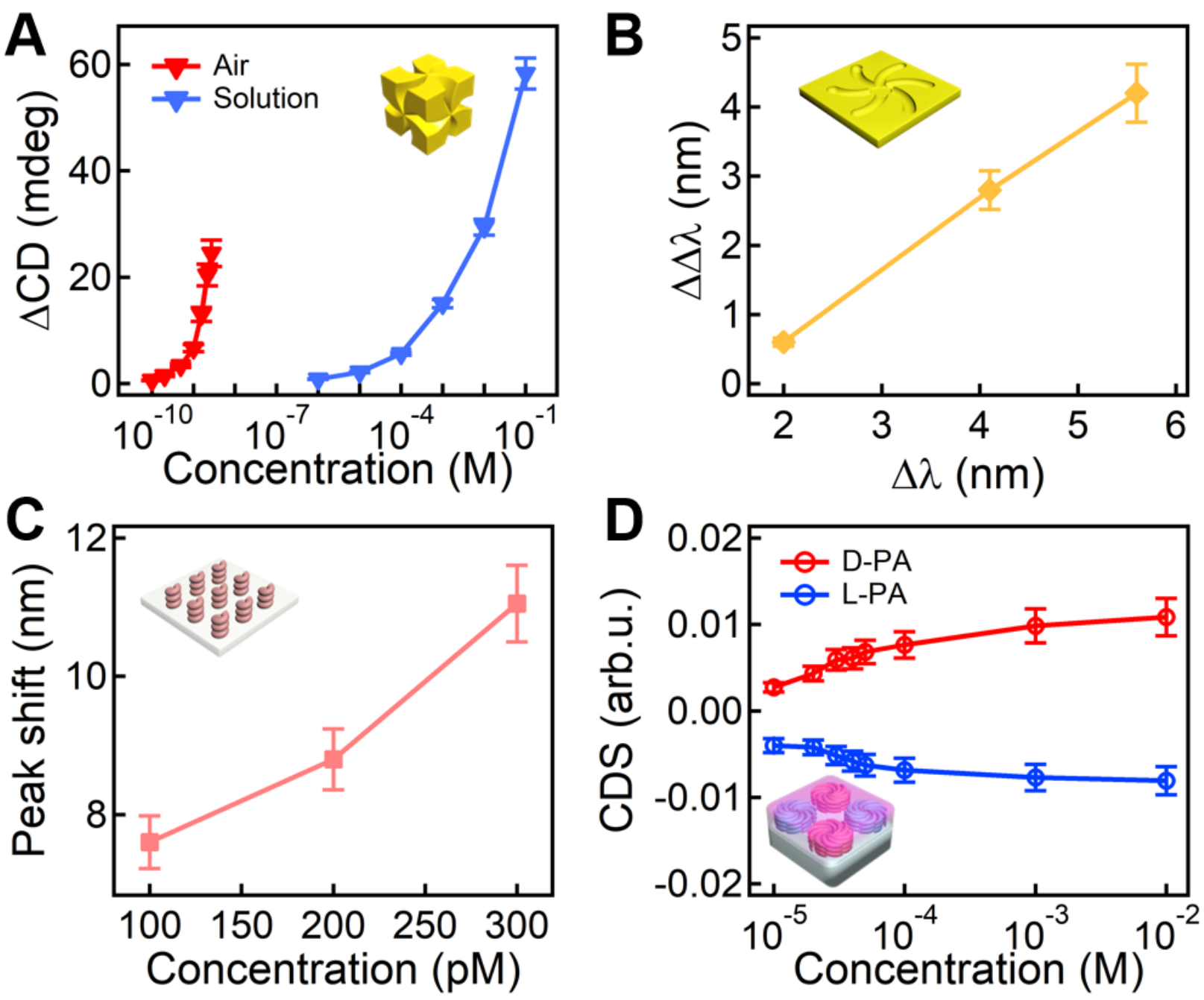

Fig. S20. **The raw data for calculating the FoM of different chiral plasmonic sensors.** (A) Au helicoid nanoarrays(*26*). (B) Shuriken structures(*19*). (C) Ag nanohelicoids(*23*). (D) Spiral Au racemic arrays(*27*).

7. Nonlinear Chiroptic Quantification of Enantiomer Purity Based on Spiral Au Metastructures

We validate the quantification of the racemates by measuring the pure enantiomers separately (Fig. S21). We mixed 100 μM L-PA and D-PA to get a racemate, which reasonably shows zero CP response (Fig. S21A). However, when we detect their chiral SHG spectra, they can substantially reduce the intrinsic difference of LCP and RCP SHG signals of the spiral Au metastructures (Fig. S21B, C), which results in a prominent ΔCP_{SHG} response (solid lines in Fig. S21F). We have also measured the ΔCP_{SHG} spectra of 100 μM L-PA and D-PA enantiomer solution separately with LH and RH spiral Au metastructures (Fig. S21D, E) and averaged them as if they were racemates (dashed lines in Fig. S21F). We found they agree well with the ΔCP_{SHG} spectra measured in the real racemates, suggesting the high selectivity and consistency of this nonlinear chiroptic sensing, which is reliable for enantiomer purity quantification.

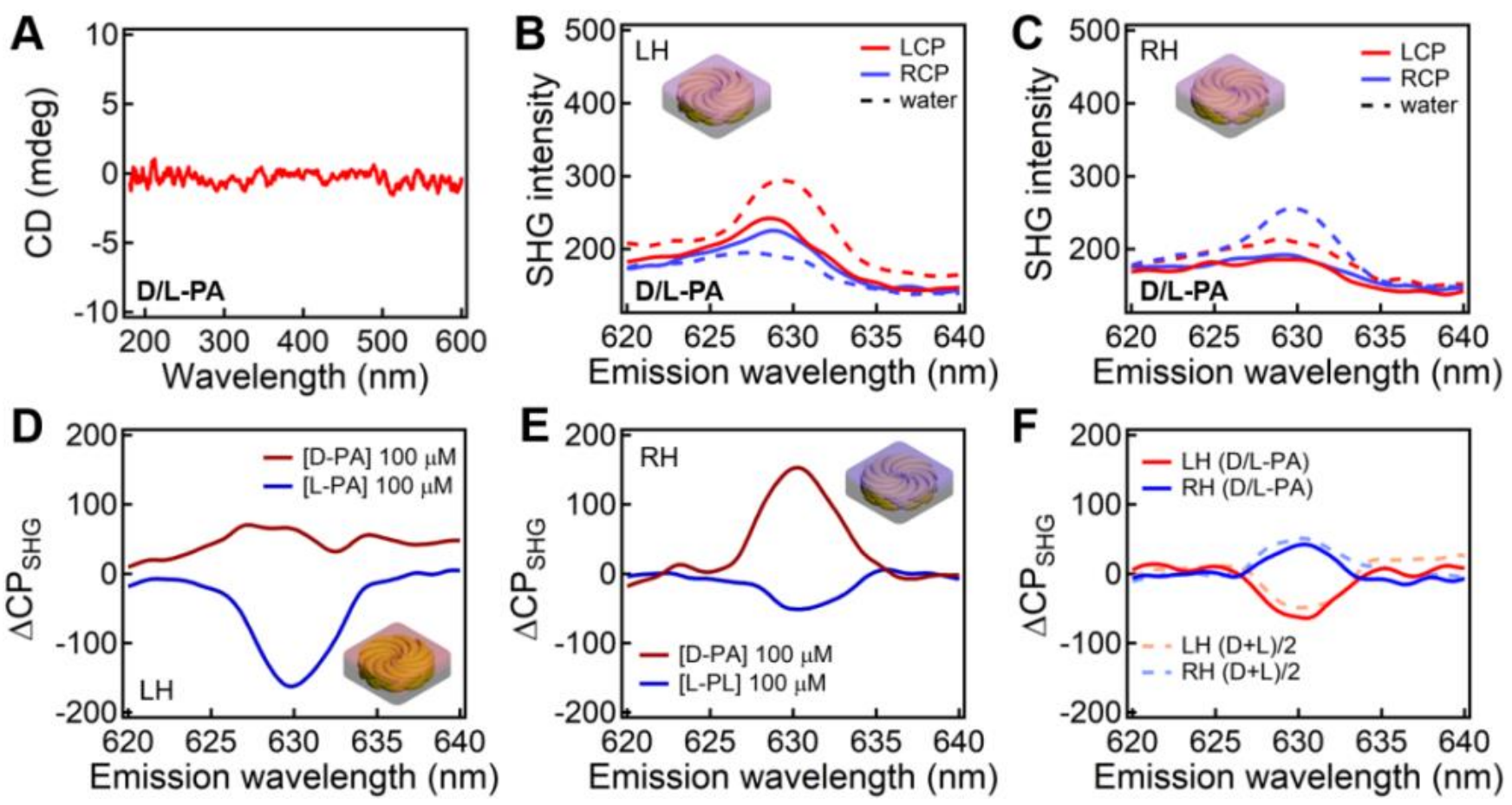

Fig. S21. **Racemate sensing with nonlinear chiroptic response of spiral Au metastructures.** (A) Linear CD spectrum of the racemic mixture of D/L-PA. Chiral SHG spectra of (B) LH and (C) RH spiral Au metastructures with (solid lines) and without (dashed lines) the presence of the racemates of D/L-PA (100 μM). ΔCP_{SHG} spectra of (D) LH and (E) RH spiral Au metastructures with the presence of enantiopure L-PA and D-PA solutions (100 μM). (F) ΔCP_{SHG} spectra of RH and LH spiral Au metastructures with the presence of racemates (solid lines) and the averaged ΔCP_{SHG} spectra from enantiopure D-PA and L-PA solutions detected with the LH and RH spiral Au metastructures (dashed lines) separately.

Such enantioselective detection is mainly due to a molecular coupling mechanism rather than preferential adsorption. Preferential adsorption typically requires chiral facets or chiral ligands, neither of which is present in our laser-written spiral Au structures. Moreover, the racemate response agrees with the average of the two enantiopure responses, consistent with independent superposition rather than competitive binding. We therefore propose that the enantioselectivity likely originates from phase matching between the chiral charge transfer within the molecule and the geometric phase of the superchiral near-field, which modulates the CP_{SHG} intensity in an enantiomer-dependent manner. Further investigation of this mechanism will be the focus of future work.

8. Setup Details for Spiral Au Metastructure Fabrication and Linear Chiroptic Characterizations

We used a 457 nm CW laser for the fabrication and CPL excitation based on the same home-built optical microscope setup system (Fig. S22). Specifically, a pair of half-waveplate and vortex retarder (VR) module was placed in the optical path of the laser beam to adjust the polarization profiles with different alignment angles (see the dashed blue box at the right bottom). For the CDS characterization, a pair of linear polarizer and quarter-waveplate (see the dashed red box) was placed after the Halogen lamp to generate LCP and RCP white light incidences on the samples to measure their chiral scattering spectra. In this case, a 50× objective was used. For the CPL characterization, a pair of switchable circular polarizer and long pass filter (see the dashed yellow box) was placed in the spectral collection optical path before the spectrometer

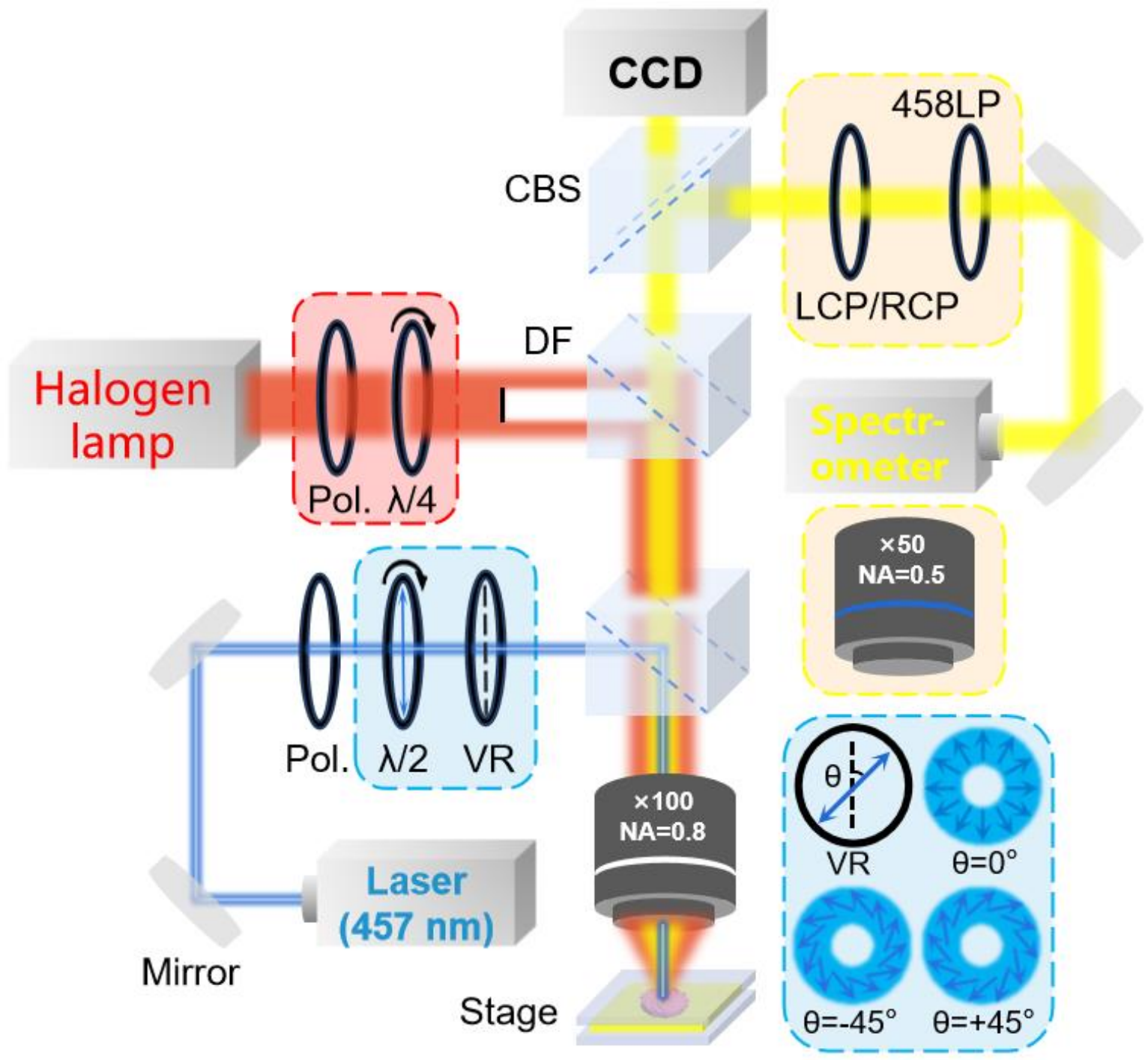

Fig. S22. Schematic setups of the laser fabrication (blue dashed box), CDS spectroscopy (red dashed box), and CPL (yellow dashed box) spectroscopy. Pol.: polarizer. λ/2: half-wave plate. λ/4: quarter-wave plate. VR: vortex retarder. LP: long pass filters. LCP/RCP: circular polarizer. DF: dark field. CBS: cube beam splitter. θ: angle between polarization direction and VR fast axis.

9. System calibration

In general, reflections from optical surfaces can induce elliptical distortion or alter the handedness of circular polarization, which could affect polarization-dependent measurements. To exclude this artefact, we performed the measurements with a flat gold film as a blank reference, which shows identical SHG signals from the LCP and RCP channels (Fig. S23). Furthermore, their sum equals to the total SHG signal collected without a polarization analyzer. This blank reference measurement suggests that the potential polarization artifacts in the optical path can be ignored.

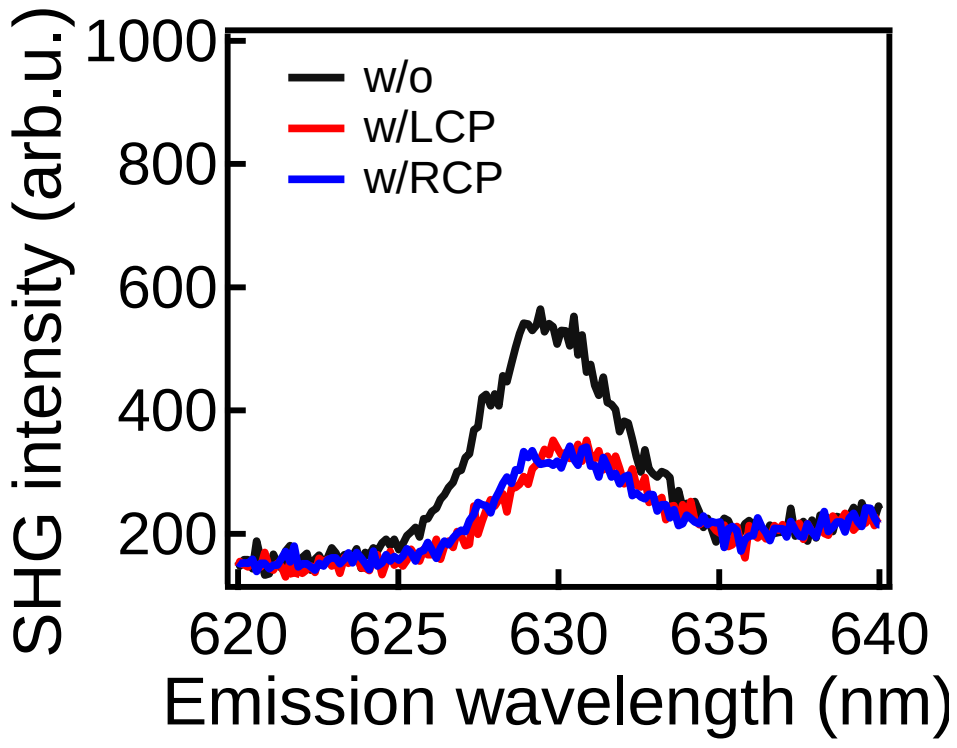

Fig. S23. SHG spectra of a flat gold film collected with or without LCP/RCP polarizer.

References

73. Z. Mao, R. Espinoza, A. Garcia, A. Enwright, H. Vang, S. C. Nguyen, Tuning Redox Potential of Gold Nanoparticle Photocatalysts by Light. *ACS Nano* **14**, 7038-7045 (2020).

74. S. Yang, Y. Wang, Q. Wang, R. Zhang, B. Ding, UV irradiation induced formation of Au nanoparticles at room temperature: The case of pH values. *Colloids Surf. Physicochem. Eng. Aspects* **301**, 174-183 (2007).

75. Y. Tang, A. E. Cohen, Optical Chirality and Its Interaction with Matter. *Phys. Rev. Lett.* **104**, 163901 (2010).

76. Y. Tang, A. E. Cohen, Enhanced Enantioselectivity in Excitation of Chiral Molecules by Superchiral Light. *Science* **332**, 333-336 (2011).

REFERENCES

1. L. Xu, X. Wang, W. Wang, M. Sun, W. J. Choi, J.-Y. Kim, C. Hao, S. Li, A. Qu, M. Lu, X. Wu, F. M. Colombari, W. R. Gomes, A. L. Blanco, A. F. de Moura, X. Guo, H. Kuang, N. A. Kotov, C. Xu, Enantiomer-dependent immunological response to chiral nanoparticles. *Nature* **601**, 366–373 (2022).

2. H.-U. Blaser, Chirality and its implications for the pharmaceutical industry. *Rend. Lincei* **24**, 213–216 (2013).

3. Y. Chen, W. Du, Q. Zhang, O. Ávalos-Ovando, J. Wu, Q.-H. Xu, N. Liu, H. Okamoto, A. O. Govorov, Q. Xiong, C.-W. Qiu, Multidimensional nanoscopic chiroptics. *Nat. Rev. Phys.* **4**, 113–124 (2022).

4. J. T. Collins, C. Kuppe, D. C. Hooper, C. Sibilia, M. Centini, V. K. Valev, Chirality and chiroptical effects in metal nanostructures: Fundamentals and current trends. *Adv. Opt. Mater.* **5**, 1700182 (2017).

5. H. G. Kuball, "Chiroptical analysis," in *Encyclopedia of Analytical Science*, P. Worsfold, C. Poole, A. Townshend, M. Miró, Eds. (Academic Press, ed. 3, 2013), pp. 1–21.

6. W. Ma, M. Sun, P. Fu, S. Li, L. Xu, H. Kuang, C. Xu, A chiral-nanoassemblies-enabled strategy for simultaneously profiling surface glycoprotein and microRNA in living cells. *Adv. Mater.* **29**, 1703410 (2017).

7. J. Cai, C. Hao, M. Sun, W. Ma, C. Xu, H. Kuang, Chiral shell core–satellite nanostructures for ultrasensitive detection of mycotoxin. *Small* **14**, e1703931 (2018).

8. G. Wang, C. Hao, W. Ma, A. Qu, C. Chen, J. Xu, C. Xu, H. Kuang, L. Xu, Chiral plasmonic triangular nanorings with SERS activity for ultrasensitive detection of amyloid proteins in Alzheimer's disease. *Adv. Mater.* **33**, e2102337 (2021).

9. Y. Zhao, A. N. Askarpour, L. Sun, J. Shi, X. Li, A. Alù, Chirality detection of enantiomers using twisted optical metamaterials. *Nat. Commun.* **8**, 14180 (2017).

10. Y. Li, S. Lin, C. Zhang, Y. Chen, S. Zhou, L. Wang, S. Chen, T. Ding, Charge transfer plasmons enabled by supramolecular plug: From optoelectronic switching to enhanced chiral sensing. *J. Am. Chem. Soc.* **146**, 28739–28747 (2024).

11. S. R. K. C. Indukuri, C. Frydendahl, N. Sharma, N. Mazurski, Y. Paltiel, U. Levy, Enhanced chiral sensing at the few-molecule level using negative index metamaterial plasmonic nanocuvettes. *ACS Nano* **16**, 17289–17297 (2022).

12. C. Zhang, H. Hu, C. Ma, Y. Li, X. Wang, D. Li, A. Movsesyan, Z. Wang, A. Govorov, Q. Gan, T. Ding, Quantum plasmonics pushes chiral sensing limit to single molecules: A paradigm for chiral biodetections. *Nat. Commun.* **15**, 2 (2024).

13. L. A. Warning, A. R. Miandashti, L. A. McCarthy, Q. Zhang, C. F. Landes, S. Link, Nanophotonic approaches for chirality sensing. *ACS Nano* **15**, 15538–15566 (2021).

14. R. M. Kim, J. H. Han, S. M. Lee, H. Kim, Y.-C. Lim, H.-E. Lee, H.-Y. Ahn, Y. H. Lee, I. H. Ha, K. T. Nam, Chiral plasmonic sensing: From the perspective of light–matter interaction. *J. Chem. Phys.* **160**, 061001 (2024).

15. M. Hwang, H. Jung, J.-Y. Kim, Chirality quantification for high-performance nanophotonic biosensors. *Small Methods* **9**, 2500112 (2025).

16. S. Im, S. Mousavi, Y.-S. Chen, Y. Zhao, Perspectives of chiral nanophotonics: From mechanisms to biomedical applications. *npj Nanophotonics* **1**, 46 (2024).

17. E. Hendry, T. Carpy, J. Johnston, M. Popland, R. V. Mikhaylovskiy, A. J. Lapthorn, S. M. Kelly, L. D. Barron, N. Gadegaard, M. Kadodwala, Ultrasensitive detection and characterization of biomolecules using superchiral fields. *Nat. Nanotechnol.* **5**, 783–787 (2010).

18. A. S. Karimullah, C. Jack, R. Tullius, V. M. Rotello, G. Cooke, N. Gadegaard, L. D. Barron, M. Kadodwala, Disposable plasmonics: Plastic templated plasmonic metamaterials with tunable chirality. *Adv. Mater.* **27**, 5610–5616 (2015).

19. R. Tullius, A. S. Karimullah, M. Rodier, B. Fitzpatrick, N. Gadegaard, L. D. Barron, V. M. Rotello, G. Cooke, A. Lapthorn, M. Kadodwala, "Superchiral" spectroscopy: Detection of protein higher order hierarchical structure with chiral plasmonic nanostructures. *J. Am. Chem. Soc.* **137**, 8380–8383 (2015).

20. X. Lu, X. Wang, S. Wang, T. Ding, Polarization-directed growth of spiral nanostructures by laser direct writing with vector beams. *Nat. Commun.* **14**, 1422 (2023).

21. Y. Wang, H. Hu, J. Tang, S. Meng, H. Xu, T. Ding, Plasmon-directed on-wire growth of branched silver nanowires with chiroptic activity. *ACS Nano* **15**, 16404–16410 (2021).

22. S. Wang, T. Ding, Laser-directed asymmetric growth of plasmonic chiral ensembles. *Laser Photonics Rev.* **16**, 2100526 (2022).

23. J.-Y. Kim, C. McGlothin, M. Cha, Z. J. Pfaffenberger, E. S. Turali Emre, W. Choi, S. Kim, J. S. Biteen, N. A. Kotov, Direct-write 3D printing of plasmonic nanohelicoids by circularly polarized light. *Proc. Natl. Acad. Sci. U.S.A.* **121**, e2312082121 (2024).

24. P. T. Probst, M. Mayer, V. Gupta, A. M. Steiner, Z. Zhou, G. K. Auernhammer, T. A. F. König, A. Fery, Mechano-tunable chiral metasurfaces via colloidal assembly. *Nat. Mater.* **20**, 1024–1028 (2021).

25. J. Li, M. Wang, Z. Wu, H. Li, G. Hu, T. Jiang, J. Guo, Y. Liu, K. Yao, Z. Chen, J. Fang, D. Fan, B. A. Korgel, A. Alù, Y. Zheng, Tunable chiral optics in all-solid-phase reconfigurable dielectric nanostructures. *Nano Lett.* **21**, 973–979 (2021).

26. R. M. Kim, J.-H. Huh, S. Yoo, T. G. Kim, C. Kim, H. Kim, J. H. Han, N. H. Cho, Y.-C. Lim, S. W. Im, E. Im, J. R. Jeong, M. H. Lee, T.-Y. Yoon, H.-Y. Lee, Q. H. Park, S. Lee, K. T. Nam, Enantioselective sensing by collective circular dichroism. *Nature* **612**, 470–476 (2022).

27. Y. Tan, X. Lu, T. Ding, Trace-amount detection of chiral molecules based on plasmonic racemic arrays fabricated via direct laser writing. *ACS Sens.* **9**, 3290–3295 (2024).

28. J. García-Guirado, M. Svedendahl, J. Puigdollers, R. Quidant, Enantiomer-selective molecular sensing using racemic nanoplasmonic arrays. *Nano Lett.* **18**, 6279–6285 (2018).

29. D. Li, G. Wang, M. Sun, A. Qu, C. Xu, Q. Wang, C. Hao, H. Kuang, Chiral bimetallic Pt@ Au octapods with spiral four-petal flower-like symmetric configuration as sensitive SERS probes. *Angew. Chem. Int. Ed.* **64**, e202501894 (2025).

30. C. Hao, D. Meng, W. Shi, C. Xu, Q. Wang, H. Kuang, Chiral gold nanostructure monolayers as SERS substrates for ultrasensitive detection of enantiomer biomarkers of Alzheimer's disease. *Angew. Chem. Int. Ed.* **64**, e202502115 (2025).

31. V. K. Valev, J. J. Baumberg, C. Sibilia, T. Verbiest, Chirality and chiroptical effects in plasmonic nanostructures: Fundamentals, recent progress, and outlook. *Adv. Mater.* **25**, 2517–2534 (2013).

32. L. M. Haupert, G. J. Simpson, Chirality in nonlinear optics. *Annu. Rev. Phys. Chem.* **60**, 345–365 (2009).

33. G. Albrecht, S. Kaiser, H. Giessen, M. Hentschel, Refractory plasmonics without refractory materials. *Nano Lett.* **17**, 6402–6408 (2017).

34. M. Kauranen, A. V. Zayats, Nonlinear plasmonics. *Nat. Photonics* **6**, 737–748 (2012).

35. S. Shen, L. Meng, Y. Zhang, J. Han, Z. Ma, S. Hu, Y. He, J. Li, B. Ren, T.-M. Shih, Z. Wang, Z. Yang, Z. Tian, Plasmon-enhanced second-harmonic generation nanorulers with ultrahigh sensitivities. *Nano Lett.* **15**, 6716–6721 (2015).

36. J. Butet, O. J. F. Martin, Nonlinear plasmonic nanorulers. *ACS Nano* **8**, 4931–4939 (2014).

37. E. M. Kim, S. S. Elovikov, T. V. Murzina, A. A. Nikulin, O. A. Aktsipetrov, M. A. Bader, G. Marowsky, Surface-enhanced optical third-harmonic generation in Ag island films. *Phys. Rev. Lett.* **95**, 227402 (2005).

38. Y. Zhang, N. K. Grady, C. Ayala-Orozco, N. J. Halas, Three-dimensional nanostructures as highly efficient generators of second harmonic light. *Nano Lett.* **11**, 5519–5523 (2011).

39. J. Butet, P.-F. Brevet, O. J. F. Martin, Optical second harmonic generation in plasmonic nanostructures: From fundamental principles to advanced applications. *ACS Nano* **9**, 10545–10562 (2015).

40. M. Celebrano, X. Wu, M. Baselli, S. Großmann, P. Biagioni, A. Locatelli, C. De Angelis, G. Cerullo, R. Osellame, B. Hecht, L. Duò, F. Ciccacci, M. Finazzi, Mode matching in multiresonant plasmonic nanoantennas for enhanced second harmonic generation. *Nat. Nanotechnol.* **10**, 412–417 (2015).

41. H. Linnenbank, Y. Grynko, J. Förstner, S. Linden, Second harmonic generation spectroscopy on hybrid plasmonic/dielectric nanoantennas. *Light Sci. Appl.* **5**, e16013 (2016).

42. Y.-H. Qiu, S.-J. Ding, Y.-J. Lin, K. Chen, D.-J. Yang, S. Ma, X. Li, H.-q. Lin, J. Wang, Q.-Q. Wang, Growth of Au hollow stars and harmonic excitation energy transfer. *ACS Nano* **14**, 736–745 (2020).

43. C.-C. Zhang, J.-Y. Zhang, J.-R. Feng, S.-T. Liu, S.-J. Ding, L. Ma, Q.-Q. Wang, Plasmon-enhanced second harmonic generation of metal nanostructures. *Nanoscale* **16**, 5960–5975 (2024).

44. M. Mesch, B. Metzger, M. Hentschel, H. Giessen, Nonlinear plasmonic sensing. *Nano Lett.* **16**, 3155–3159 (2016).

45. L. Ghirardini, A.-L. Baudrion, M. Monticelli, D. Petti, P. Biagioni, L. Duò, G. Pellegrini, P.-M. Adam, M. Finazzi, M. Celebrano, Plasmon-enhanced second harmonic sensing. *J. Phys. Chem. C* **122**, 11475–11481 (2018).

46. M. S. Verma, M. Chandra, Second harmonic generation-based nonlinear plasmonic RI-sensing in solution: The pivotal role of the particle size. *Phys. Chem. Chem. Phys.* **23**, 25565–25571 (2021).

47. Z. Zheng, D. Rocco, H. Ren, O. Sergaeva, Y. Zhang, K. B. Whaley, C. Ying, D. d. Ceglia, C. De-Angelis, M. Rahmani, L. Xu, Advances in nonlinear metasurfaces for imaging, quantum, and sensing applications. *Nanophotonics* **12**, 4255–4281 (2023).

48. A. Rodriguez, M. Soljačić, J. D. Joannopoulos, S. G. Johnson, $\chi^{(2)}$ and $\chi^{(3)}$ harmonic generation at a critical power in inhomogeneous doubly resonant cavities. *Opt. Express* **15**, 7303–7318 (2007).

49. S. Arnold, M. Khoshsima, I. Teraoka, S. Holler, F. Vollmer, Shift of whispering-gallery modes in microspheres by protein adsorption. *Opt. Lett.* **28**, 272–274 (2003).

50. G. Serrera, J. González-Colsa, P. Albella, Amplified linear and nonlinear chiral sensing assisted by anapole modes in hybrid metasurfaces. *Appl. Phys. Lett.* **124**, 251701 (2024).

51. S. Sharma, A. Karabchevsky, S. K. Varshney, Accidental BICs and chiroptical response of hollow channels in silicon nanodisk resonator-based metasurfaces for chiral sensing. *ACS Appl. Opt. Mater.* **3**, 364–374 (2025).

52. S. P. Rodrigues, S. Lan, L. Kang, Y. Cui, W. Cai, Nonlinear imaging and spectroscopy of chiral metamaterials. *Adv. Mater.* **26**, 6157–6162 (2014).

53. S. P. Rodrigues, Y. Cui, S. Lan, L. Kang, W. Cai, Metamaterials enable chiral-selective enhancement of two-photon luminescence from quantum emitters. *Adv. Mater.* **27**, 1124–1130 (2015).

54. G. Li, S. Zhang, T. Zentgraf, Nonlinear photonic metasurfaces. *Nat. Rev. Mater.* **2**, 17010 (2017).

55. V. K. Valev, J. J. Baumberg, B. De Clercq, N. Braz, X. Zheng, E. J. Osley, S. Vandendriessche, M. Hojeij, C. Blejean, J. Mertens, C. G. Biris, V. Volskiy, M. Ameloot, Y. Ekinci, G. A. E. Vandenbosch, P. A. Warburton, V. V. Moshchalkov, N. C. Panoiu, T. Verbiest, Nonlinear superchiral meta-surfaces: Tuning chirality and disentangling non-reciprocity at the nanoscale. *Adv. Mater.* **26**, 4074–4081 (2014).

56. L. Ohnoutek, J.-Y. Kim, J. Lu, B. J. Olohan, D. M. Răsădean, G. Dan Pantoș, N. A. Kotov, V. K. Valev, Third-harmonic Mie scattering from semiconductor nanohelices. *Nat. Photonics* **16**, 126–133 (2022).

57. B. J. Olohan, E. Petronijevic, U. Kilic, S. Wimer, M. Hilfiker, M. Schubert, C. Argyropoulos, E. Schubert, S. R. Clowes, G. D. Pantoş, D. L. Andrews, V. K. Valev, Chiroptical second-harmonic Tyndall scattering from silicon nanohelices. *ACS Nano* **18**, 16766–16775 (2024).

58. C. Jack, A. S. Karimullah, R. Leyman, R. Tullius, V. M. Rotello, G. Cooke, N. Gadegaard, L. D. Barron, M. Kadodwala, Biomacromolecular stereostructure mediates mode hybridization in chiral plasmonic nanostructures. *Nano Lett.* **16**, 5806–5814 (2016).

59. O. Neufeld, O. Cohen, Optical chirality in nonlinear optics: Application to high harmonic generation. *Phys. Rev. Lett.* **120**, 133206 (2018).

60. M. S. Verma, M. Chandra, Nonlinear plasmonic sensing for label-free and selective detection of mercury at picomolar level. *ACS Sens.* **5**, 645–649 (2020).

61. S. Yoo, Q.-H. Park, Nanophotonic chiral sensing: From principles to practice. *Photonics Insights* **4**, R08 (2025).

62. M. Ren, E. Plum, J. Xu, N. I. Zheludev, Giant nonlinear optical activity in a plasmonic metamaterial. *Nat. Commun.* **3**, 833 (2012).

63. L. Gui, M. Hentschel, J. Defrance, J. Krauth, T. Weiss, H. Giessen, Nonlinear Born-Kuhn analog for chiral plasmonics. *ACS Photonics* **6**, 3306–3314 (2019).

64. F. Spreyer, C. Ruppert, P. Georgi, T. Zentgraf, Influence of plasmon resonances and symmetry effects on second harmonic generation in WS_2–plasmonic hybrid metasurfaces. *ACS Nano* **15**, 16719–16728 (2021).

65. F. Spreyer, J. Mun, H. Kim, R. M. Kim, K. T. Nam, J. Rho, T. Zentgraf, Second harmonic optical circular dichroism of plasmonic chiral helicoid-III nanoparticles. *ACS Photonics* **9**, 784–792 (2022).

66. G. Saerens, G. Ellrott, O. Pashina, I. Deriy, V. Krstić, M. Petrov, M. Chekhova, R. Grange, Second-order nonlinear circular dichroism in square lattice array of germanium nanohelices. *ACS Photonics* **11**, 3630–3635 (2024).

67. D. Gromyko, J. S. Loh, J. Feng, C.-W. Qiu, L. Wu, Enabling all-to-circular polarization up-conversion by nonlinear chiral metasurfaces with rotational symmetry. *Phys. Rev. Lett.* **134**, 023804 (2025).

68. Y. Tan, Y. Chen, X. Lu, Z. Dang, Z. Fang, T. Ding, Polarization-directed construction of 3D spiral plasmonic micropillars for enhanced chiral photoluminescence. *Laser Photonics Rev.* **19**, e01268 (2025).

69. L. Novotny, B. Hecht, *Principles of Nano-Optics* (Cambridge Univ. Press, ed. 2, 2012).

70. R. D. Johnson, F. H. Arnold, The temkin isotherm describes heterogeneous protein adsorption. *Biochim. Biophys. Acta* **1247**, 293–297 (1995).

71. G. Bachelier, J. Butet, I. Russier-Antoine, C. Jonin, E. Benichou, P. F. Brevet, Origin of optical second-harmonic generation in spherical gold nanoparticles: Local surface and nonlocal bulk contributions. *Phys. Rev. B* **82**, 235403 (2010).

72. A. Biswas, P. Cencillo-Abad, M. W. Shabbir, M. Karmakar, D. Chanda, Tunable plasmonic superchiral light for ultrasensitive detection of chiral molecules. *Sci. Adv.* **10**, eadk2560 (2024).

73. Z. Mao, R. Espinoza, A. Garcia, A. Enwright, H. Vang, S. C. Nguyen, Tuning redox potential of gold nanoparticle photocatalysts by light. *ACS Nano* **14**, 7038–7045 (2020).

74. S. Yang, Y. Wang, Q. Wang, R. Zhang, B. Ding, UV irradiation induced formation of Au nanoparticles at room temperature: The case of pH values. *Colloids Surf. A Physicochem. Eng. Asp.* **301**, 174–183 (2007).

75. Y. Tang, A. E. Cohen, Optical chirality and its interaction with matter. *Phys. Rev. Lett.* **104**, 163901 (2010).

76. Y. Tang, A. E. Cohen, Enhanced enantioselectivity in excitation of chiral molecules by superchiral light. *Science* **332**, 333–336 (2011).
